# Evolution of Gene Expression in the Uterine Cervix related to Steroid Signaling: Conserved features in the regulation of cervical ripening

**DOI:** 10.1038/s41598-017-04759-6

**Published:** 2017-06-30

**Authors:** Günter P. Wagner, Mauris C. Nnamani, Arun Rajendra Chavan, Jamie Maziarz, Stella Protopapas, Jennifer Condon, Roberto Romero

**Affiliations:** 1 Department of Ecology and Evolutionary Biology, Yale University, New Haven, CT 06520 USA; 2 Department of Obstetrics, Gynecology and Reproductive Science, Yale University, New Haven, CT 06520 USA; 3 Yale Systems Biology Institute, West Haven, CT 06516 USA; 4 Perinatology Research Branch, NICHD, NIH, Detroit, MI 48201 USA; 5 Department of Obstetrics and Gynecology, Wayne State University, Detroit, MI 48202 USA; 6 Department of Obstetrics and Gynecology, University of Michigan, Ann Arbor, MI 48109 USA; 7 Department of Epidemiology, Michigan State University, East Lansing, MI 48824 USA

## Abstract

The uterine cervix is the boundary structure between the uterus and the vagina and is key for the maintenance of pregnancy and timing of parturition. Here we report on a comparative transcriptomic study of the cervix of four placental mammals, mouse, guinea pig, rabbit and armadillo, and one marsupial, opossum. Our aim is to investigate the evolution of cervical gene expression as related to putative mechanisms for functional progesterone withdrawal. Our findings are: 1) The patterns of gene expression in eutherian (placental) mammals are consistent with the notion that an increase in the E/P4 signaling ratio is critical for cervical ripening. How the increased E/P4 ratio is achieved, however, is variable between species. 2) None of the genes related to steroid signaling, that are modulated in eutherian species, change expression during opossum gestation. 3) A tendency for decreased expression of progesterone receptor co-activators (NCOA1, -2 and -3, and CREBBP) towards term is a shared derived feature of eutherians. This suggests that parturition is associated with broad scale histone de-acetylation. Western-blotting on mouse cervix confirmed large scale histone de-acetylation in labor. This finding may have important implications for the control of premature cervical ripening and prevention of preterm birth in humans.

## Introduction

Pregnancy of eutherian mammals is characterized by an extensive period of gestation, maternal recognition of pregnancy, and often deeply invasive placentation^1–5^. Here we study the origin of eutherian pregnancy by investigating gene expression in a key component of the female reproductive tract, the uterine cervix, a key organ for the maintenance of human pregnancy^6–11^. Premature shortening of the cervix is the strongest clinical predictor of premature birth^10^. It is also critically important for making term delivery possible in humans^10^, mice^8^, sheep^12^, cow^13, 14^. How cervical remodeling is regulated is one of the major unanswered questions in female reproductive biology and critical for research on the prevention of prematurity^15^. In this study we focus on genes related to progesterone (P4) and estrogen (E) signaling. An increase in the E to P4 signaling ratio is associated with the onset of labor in various eutherian animals^16, 17^.

Cervical ripening is the transformation of the extracellular matrix of the uterine cervix that eventually allows its distention during the extrusion phase of parturition^6–8^. In eutherian mammals physiological cervical ripening is caused by a weakening of progesterone signaling relative to estrogen signaling through a process of functional progesterone withdrawal (FPW)^16–19^. FPW can be caused by local expression of catabolic enzymes, or the down-regulation of genes involved in the progesterone signal transduction^19, 20^. A paradigmatic example of the role of catabolic enzymes in FPW is the up-regulation of 5-α steroid reductase (SRD5A1) in the mouse cervix, which has been shown to be essential for cervical remodeling^21^. Here we present evidence that FPW likely evolved in the eutherian stem lineage and the mechanisms of FPW are consistent with the model that E/P4 ratio is critical for cervical ripening. However, the details how an increased E/P4 signaling ratio is achieved are highly species specific. There are also a few other shared derived features conserved among eutherian species. We suggest that these conserved features could be explored to influence the timing of cervical ripening in women.

## Results

**Figure 1:**
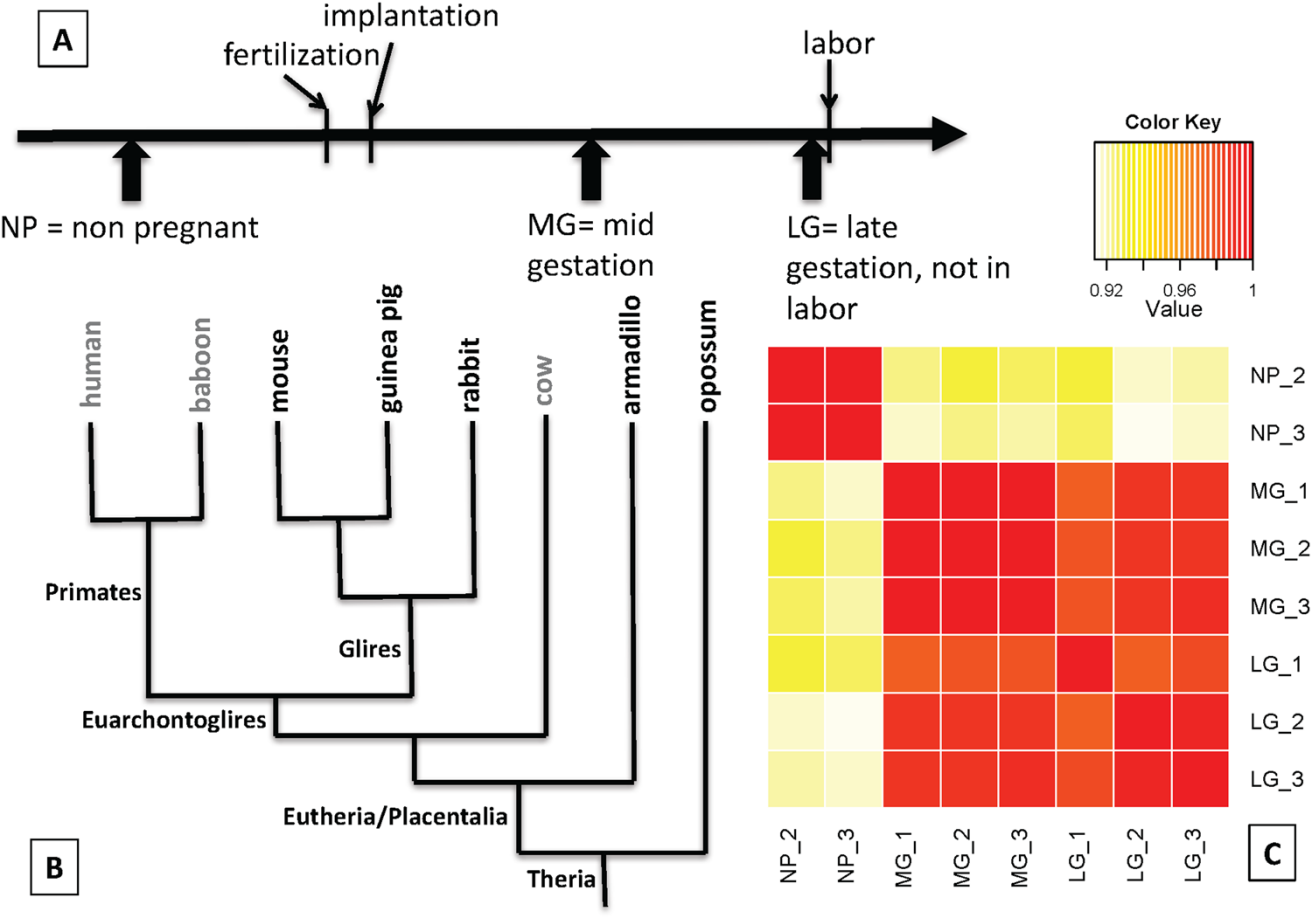
Sampling scheme for the present study. (**A**) Schematic representation of samples obtained from opossum, armadillo, rabbit, guinea pig and mouse; non-pregnant, mid gestation and last gestation samples were obtained. (**B**) Phylogenetic relationships of taxa sampled (in bold) in relation to other major groups that are not included in this study (cow and primates). (**C**) Heat map of the correlations among mouse cervical samples. Samples are labeled with the gestational stage and an index identifying the sample. The matrix represents the Pearson correlations among square root transformed TPM values. Note that the main difference is between samples from non-pregnant and pregnant animals, while the similarity among mid- and late gestation samples is quite high.

Three kinds of cervical samples were obtained: non-pregnant (NP), mid-gestation (MG) and late gestation (LG) (Fig. 1A), RNA was extracted and sequenced. Samples were obtained at least in triplicate with the exception of late gestation armadillo, for which only one animal was collected in the field. Samples of poor quality were identified by comparison with other samples from the same species and eliminated before further analysis. Figure 1C shows the similarity of samples from the mouse indicating that the strongest difference exists between samples from non-pregnant animals and those taken during pregnancy.

There is a consensus that progesterone (P4) signaling is essential for the maintenance of pregnancy in eutherian mammals^19, 22–24^. There is also a consensus that withdrawal of progesterone signaling is important for cervical ripening^6–8^. Progesterone withdrawal can happen systemically, by decrease of P4 production, increase in local P4 destruction by catabolic enzymes, decrease of P4 receptor (PGR) expression or decreased expression of activating PGR co-factors. Below we will first start with genes coding for catabolic enzymes of P4.

### Genes related to progesterone catabolism

There are two enzymatic reactions documented from the female reproductive tract that lead to the removal of P4. These are the reduction of the 4–5 double bond in the A-ring of the steroid scaffold by 5α-steroid reductase type 1, SRD5A1^21^, and the reduction of the C20 keto-group producing 20α OH-progesterone by 20α OH steroid reductase activity, 20α HSD^25–27^, a member of the aldo-keto-reductase protein family 1.

**Figure 2:**
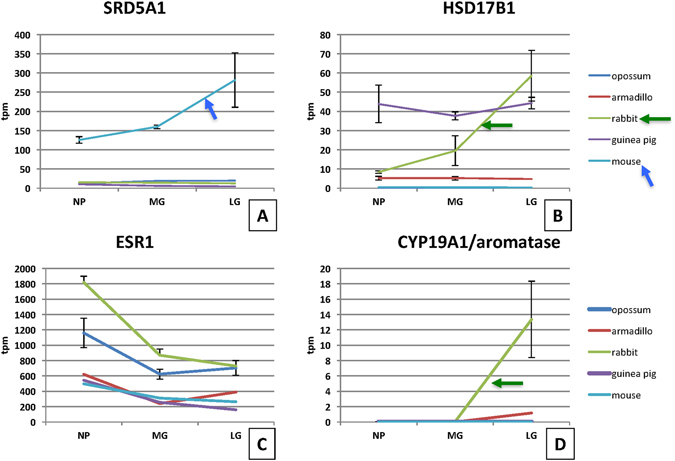
mRNA expression profiles of some genes related to progesterone and estrogen metabolism. (**A**) SRD5A1 is an enzyme that converts progesterone to di-hydroprogesterone and is necessary for mouse cervical remodeling. This gene is only expressed in the mouse in increasing amounts towards term. (**B**) HSD17B1 has been reported to have two enzymatic activities, converting estrone into estradiol, as well as hydroxylating progesterone at the C20. It is expressed in guinea pig and rabbit but only up-regulated towards term in rabbit. (**C**) The estrogen receptor alpha gene *ESR1* is expressed in all animals studied and tends to be higher in non-pregnant animals than during gestation. (**D**) *CYP19A1* is the gene for aromatase, the key enzyme for the synthesis of estrogens. This gene is not expressed in most animals with the notable exception of rabbit, which shows a strong increase towards term suggesting local production of estrogen towards term. This interpretation is further supported by the elevated expression of HSD17B1 (**B**) also uniquely in rabbit.

*SRD5A1* is lowly expressed in the uterine cervix of all species examined, with the exception of the mouse. In the mouse and only in the mouse *SRD5A1* is highly expressed at 150 to 300 TPM in late gestation samples (Kruskal-Wallis ANOVA p = 0.0439) (Fig. 2A). This is consistent with the experimental finding that *SRD5A1* is necessary for cervical ripening in mice. *SRD5A1*−/− mutants have elevated P4 levels in the cervix and the cervix fails to ripen, even though contractions ensue normally and on time^21^. Our finding that *SRD5A1* mRNA expression is only found in mice suggests that this well documented mechanism of cervical ripening is murine specific, not even found in guinea pig, which belongs to a basally branching rodent lineage^28^.

The comparative analysis of the other catabolic pathway for P4, 20α HSD, is complicated by the extensive history of gene duplication affecting the *AKR1C* gene family to which enzymes classified as 20α HSD belong^29, 30^. In addition, unrelated proteins can have 20α HSD activity, for instance HSD17B1, mostly known for its role in estradiol synthesis, has been reported to also have 20α HSD activity (http://www.genecards.org/cgi-bin/carddisp.pl?gene=HSD17B1&keywords=HSD17B1#function).

*HSD17B1* is not expressed in opossum and mouse cervix and lowly in armadillo cervix (Fig. 2B). The guinea pig has a relatively high expression in all stages of the reproductive cycle. In rabbit *HSD17B1* is highly up-regulated in late gestation (ANOVA p = 0.0104). The latter observation is probably related to local synthesis of E2 in the rabbit cervix (see below).

*AKR1C* genes encode members of a large family of enzymes called aldo-keto reductases (AKRs), and are involved in steroid, prostaglandin, and retinoid metabolism^27^. AKR1Cs catalyze NADPH dependent reduction of carbonyl groups at positions 3, 17 or 20 in steroids to form 3α/β, 17β or 20α hydoxy-steroid metabolites. In humans AKR1C1 is expressed in the cervix and converting progesterone into 20α-OHP^31^ and is induced by IL-1β and may thus be involved in inflammation induced preterm birth^32–37^.

**Figure 3:**
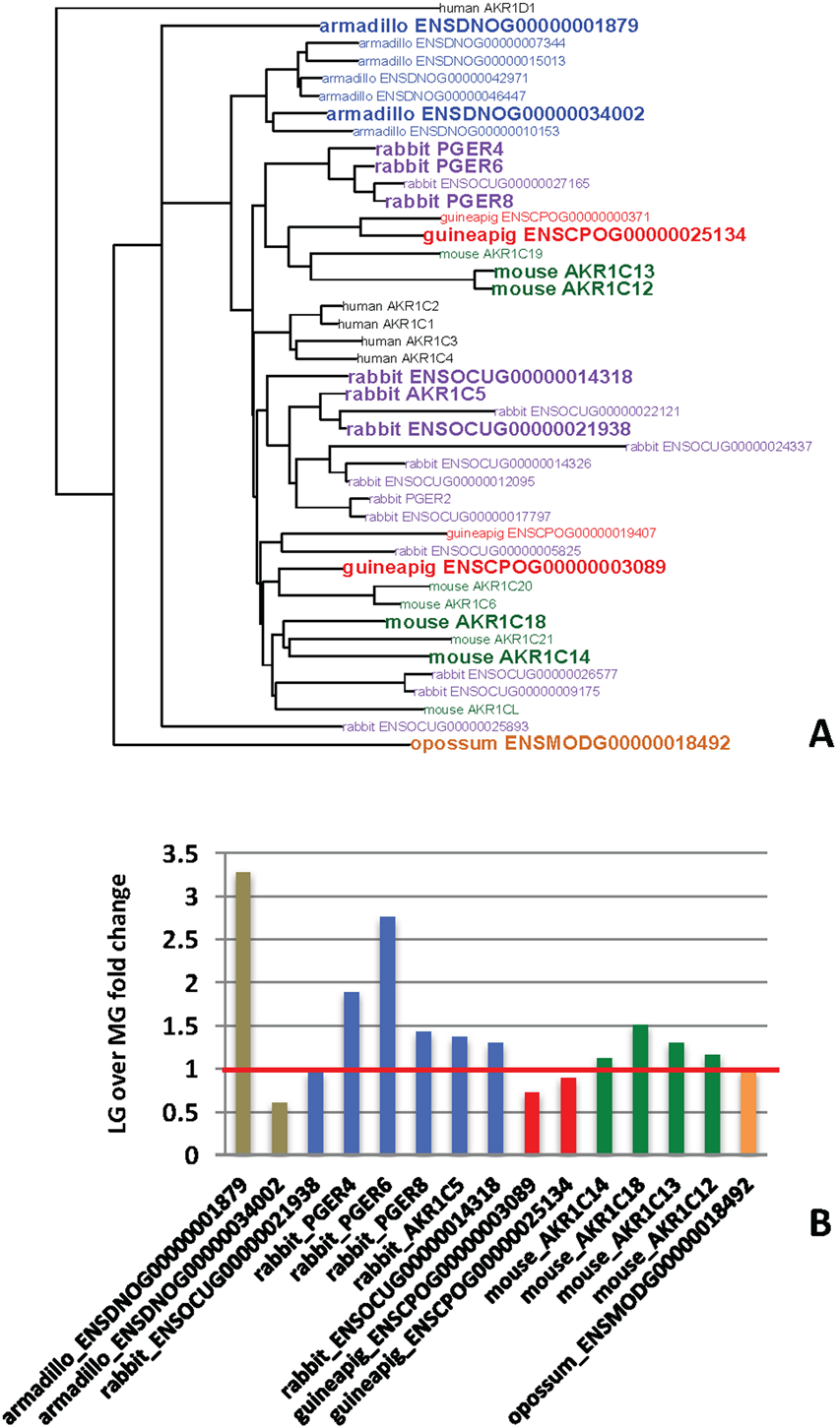
Comparative expression of aldo-keto reductase genes *AKR1C*. (**A**) The gene tree of *AKR1C1* genes. Note that there is only one *AKR1C* gene in opossum, but many independently evolved paralogs in eutherian species. Genes with expression above 3 TPM in at least one sample are in bold. (**B**) Late gestation over mid gestation fold change of *AKR1C* genes expressed in the cervix. No detectable change occurs in the opossum cervix but at least one gene experiences an increase in expression in each of the eutherian species with the exception of the guinea pig.

The gene tree of *AKR1* gene family was downloaded from ENSEMBL. This tree was pruned to only retain the *AKR1C* clade and species included in this study (and human, for reference). In Fig. 3A, the genes from the *AKR1C* clade that are expressed in the cervix above 3TPM at any stage of pregnancy are indicated in bold.

According to this tree, opossum has only one *AKR1C* gene, and it is the most basal branch in the *AKR1C* gene tree (Fig. 3A). This gene shows no modulation of expression between mid and late gestational stages (Fig. 3B). All eutherians in our dataset have more than one *AKR1C* gene, most of them due to gene duplications in the eutherian clades. Although only some of them are expressed in the cervix, the levels of all of the expressed ones are modulated during pregnancy, with the exception of guinea pig. The expression changes at the transition from mid-gestation to late-gestation are summarized in Fig. 3B. In armadillo the highest expressed *AKR1C* gene is up-regulated at late-gestation but, to our knowledge, the biochemical activity of its protein is unknown. In rabbit, the highest expressed *AKR1C* gene is ENSOCUG00000021938, a putative prostaglandin reductase (between 180 and 230 TPM). The other two highly expressed genes, PGER4 and -6, are lower in mid gestation than in non-pregnant cervix but up-regulated towards at late gestation. Both are annotated as prostaglandin E2 reductases. AKR1C18, the canonical 20α-hydroxy steroid dehydrogenase (HSD) of mouse, also increases from mid to late-gestation although only reaches moderate RNA expression levels for an enzyme (about 10 to 15 TPM).

No evidence of AKR1C mediated P4 decay (i.e. no upregulation at late gestation) was found for opossum, and guinea pig. Mouse AKR1C18 is nominally upregulated but remains relatively lowly expressed and no 20α HSD enzyme activity was previously detected in mouse cervix at term^21^. Strong up-regulation of some *AKR1C* genes in rabbit and armadillo are hard to interpret because of lack of biochemical data on the products of these genes. At the very least this data suggests that 20α HSD mediated P4 catabolism in the cervix is not a conserved feature among mammals even though it is happening in human cervix^31^.

### Genes related to estrogen signaling and metabolism

Estrogen has been identified as a key signal in cervical ripening^7^ and differences in estrogen metabolic enzyme have been documented in human cervical epithelium^31^. *ESR1* mRNA is highly expressed in all samples examined in this study and decreasing during pregnancy (Fig. 2C). We compared the expression of the genes for two key enzymes in the biosynthesis of estrogen, first *Cyp19A1*, which produces aromatase, and *HSD17B1*, which produces an enzyme that is converting estrone into estradiol, E1 → E2. Aromatase mRNA, *Cyp19A1*, has only been found in late gestation rabbit cervix samples (Fig. 2D). As mentioned above in rabbit *HSD17B1* expression is sharply increasing towards late gestation (Fig. 2B). The parallel increase of both aromatase and *HSD17B1* at late gestation in rabbit is suggestive of local synthesis of estradiol in the rabbit cervix.

An enzyme that inactivates estradiol, E2, by converting E2 into E1, estrone, is HSD17B2, which is upregulated during pregnancy in human cervical epithelium supposedly to ensure high P4/E2 ratio during pregnancy^31^. In our taxon sample transcripts of *HSD17B2* have not been detected above a threshold of 1 TPM in any sample suggesting, contrary to the situation in humans, no role of this catabolic pathway in the animals studied here. Inactivation of E2 by conversion to E1 is not a mechanism for the maintenance of cervical integrity conserved in non-human mammals, and could be derived in the primate lineage.

Other catabolic pathways are 2-hydroxylation (2-OH) and 4-hydroxylation (4-OH), by CYP1A1 and CYP1B1 respectively, and subsequent conjugation with methyl and sulfonyl groups^38^. In our data *CYP1A1* was not mapped but *CYP1B1* transcripts were mapped in all species. *CYP1B1* mRNA is highly expressed in the cervix of opossum and armadillo, but at most moderately in gestation and non-pregnant guinea pig (not shown). This data suggest that E2 breakdown through 4-hydroxylation may play a role in mammals distantly related to Glires (Glires = rodents and lagomorphs), but not in Glires.

### Genes related to progesterone signaling

**Figure 4:**
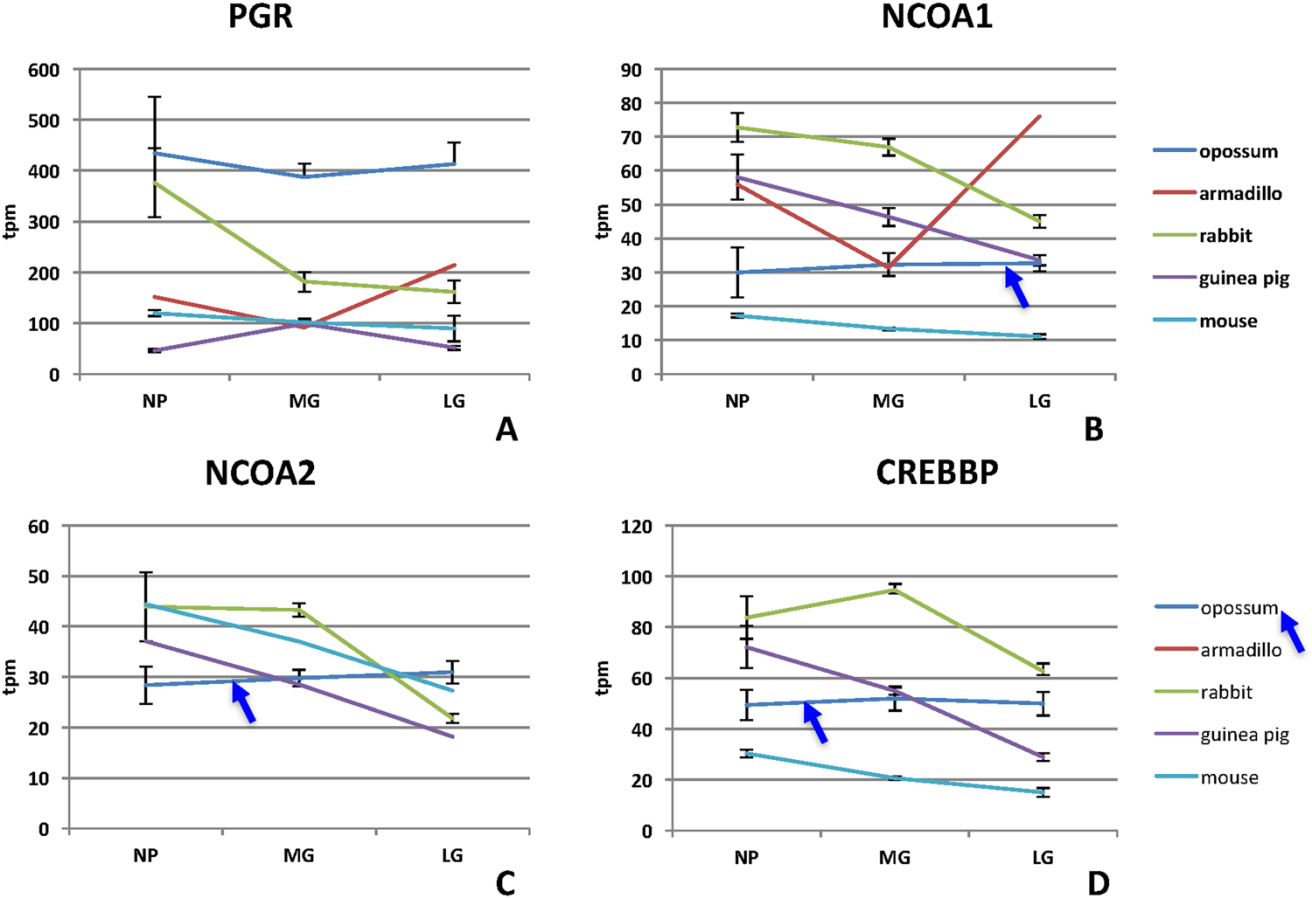
Comparative expression of genes related to progesterone signaling. (**A**) Expression profile of the progesterone receptor. (**B**) Expression profile of *NCOA1*. (**C**) Expression profile of *NCOA2*. This gene was not mapped in the armadillo genome. (**D**) Expression profile of *CREBP*. Note that these genes are not modulated in opossum and in the glires they tend to decrease in expression towards term.

Progesterone receptor (PGR) mRNA is expressed highly in all species examined, with the highest expression of 400TPM in opossum (Fig. 4A). Modulation of PGR expression consistent with functional progesterone withdrawal was only observed in the guinea pig with a 50% decline between mid gestational and late gestational samples^39^ (ANOVA p = 4.4 10^−4^). *KLF9* gene encodes a PGR interacting transcription factor^40^ with potential function in endometrium and parturition^41, 42^. *KLF9* is expressed in all species examined at or above 40 TPM (not shown). Expression profiles apparently are species specific rather than shared among groups of species. Expression remains constant between mid and late gestation in opossum and guinea pig but decreases in the mouse by a factor of 0.76 (one tailed t test p = 2.4 10^−3^). A similar decline in rabbit is not evident in our analysis.

**Figure 5:**
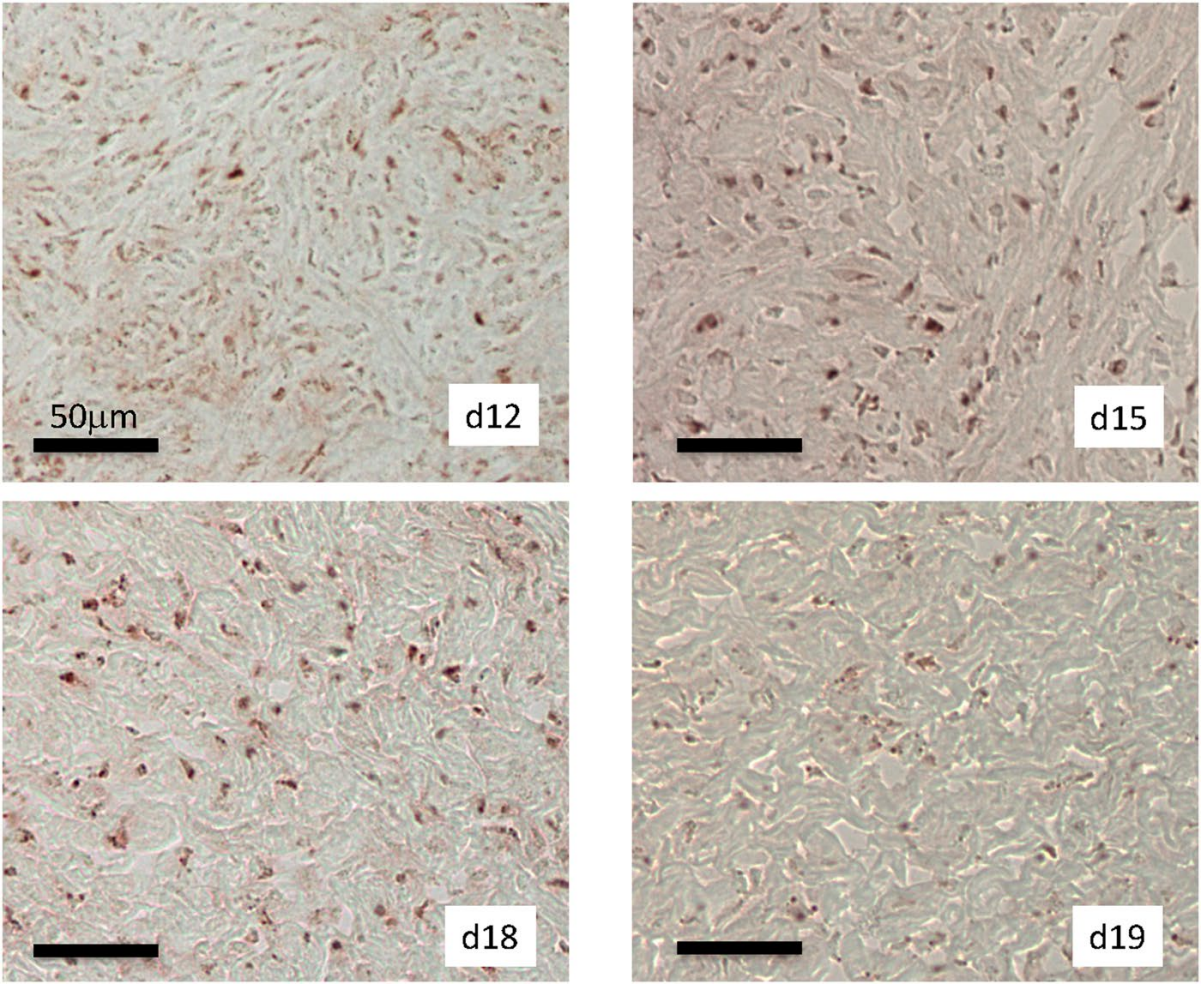
Immuno-histochemical localization of NCOA2 protein in the stroma of the posterior cervix of the mouse at days post copulation 12, 15, 18 and 19 (in labor). We show paraffin cross sections of PFA fixed material. Note the decrease of staining intensity at 19 dpc.

The only exceptions to the highly species-specific patterns of gene expression described above are some PGR co-factors. For instance *NCOA-1* as well as *NCOA-2* transcript abundance is decreasing in all three Glires species but not in opossum (Fig. 4B,C) (ANOVA, *NCOA1*: rabbit p = 9.92 10^−4^, guinea pig p = 6.4 10^−4^, mouse p = 3.4 10^−3^; *NCOA2* rabbit p = 5.9 10^−3^, guinea pig p = 3.4 10^−5^, mouse p = 1.5 10^−2^). This pattern is also found for *CREBBP* (Fig. 4D) (ANOVA, rabbit p = 1.4 10^−2^, guinea pig p = 9.0 10^−6^, mouse p = 3.2 10^−3^). Since NCOAs activate target genes by recruiting histone acetyl-transferases this pattern suggests that cervical ripening may be associated with a tendency to histone de-acetylation, as observed in the mouse and human myometrium^43^. NCOA2 protein staining is found in the nuclei of the mouse cervical stroma (Fig. 5) and the epithelium (not shown). The intensity of NCOA2 staining apparently decreases in the vaginal stroma of the cervix (Fig. 5D), but remains high in the uterine part of the cervix (Supplemental Fig. 2).

### Histone3 acetylation in the mouse cervix

**Figure 6:**
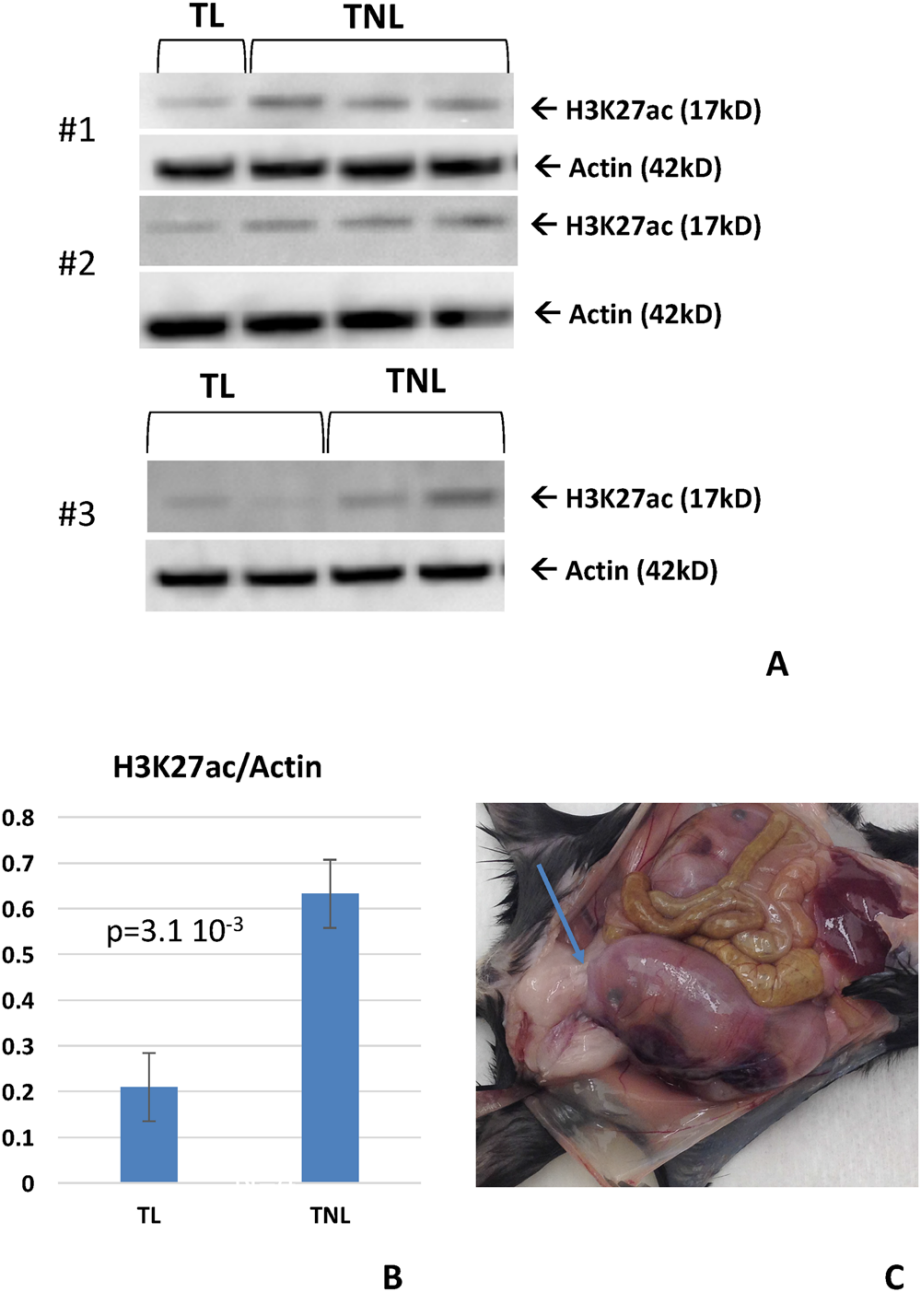
Degree of histone acetylation (H3K27ac) in the mouse cervix. (**A**) Western blots for H3K27ac, and actin at day 18 TNL (term not in labor), and 18 TL (term in labor). This panel summarizes three experiments, #1, #2, and #3. In experiments #1 and #2 each one mouse was anatomically diagnosed as in labor (TL), but before extrusion (see C for example), and experiment #3 had two animals TL. (**B**) Relative quantification of western blotting signal, H3K27ac/actin, for E18TNL and E18TL samples. Note the lower degree of histone acetylation of samples taken from animals in labor compared to term but not in labor samples. (**C**) Anatomical diagnosis of TL. One fetus in engaged with the cervix but has not yet entered the birth canal. Full gel/blotting images are provided in the supplementary material.

The level of H3K27 acetylation was compared between E16 and E18 and the degree of acetylation seems to increase from mid gestation to late gestation (Suppl. Fig. 1). We also compared E18 samples from mice in labor (TL) and not in labor (TNL). In mice labor is technically defined as the state of gestation after the first pup is born. We wanted to avoid the changes caused by the distention of the cervix and monitored mice for behavioral signs of labor (self-inspection of the vulva, stretching, restlessness) and confirmed or rejected the diagnoses of labor during necropsy. Labor was diagnosed if the first pup has moved from the uterine horn into the uterine corpus (Fig. 6C). We found that H3K27 acetylation precipitously decreases in labor (Fig. 6A,B). These findings are similar to those in the myometrium^43^ and suggest that deacetylation is associated with the onset of labor in both the myometrium as well as in the cervix.

### Relaxin and prostaglandin signaling in opossum

The apparent lack of FPW in opossum led us to investigate the expression of genes known to be important for marsupial parturition, relaxin and prostaglandin signaling. Of the four relaxin receptors only RSFP1 is expressed in the cervix of all species examined. In opossum, the expression rises from 45 TPM in NP to 250 TPM at MG and remains high also in late gestation (205 TPM, p = 0.011 ANOVA over all samples; no difference between MG and LG, p = 0.4 t-test and others). There is also a 2x increase in the expression of the PGE2 receptor 4, *PTGER4*, from 74 TPM in NP to >150 TPM in gestation (ANOVA over all samples p = 3 10^−3^). The receptor of PGF2a seems not to be annotated in the *Monodelphis* genome. This data is consistent with a model were cervical remodeling and ripening is under the influence of relaxin and prostaglandin in opossum.

## Discussion

In this paper we investigate the evolution of pregnancy with a selected set of placental mammals by focusing on one critical subsystem of the female reproductive tract, the uterine cervix. Here we will discuss our findings from both the evolutionary and functional perspective.

### Evolution of functional progesterone withdrawal

**Figure 7:**
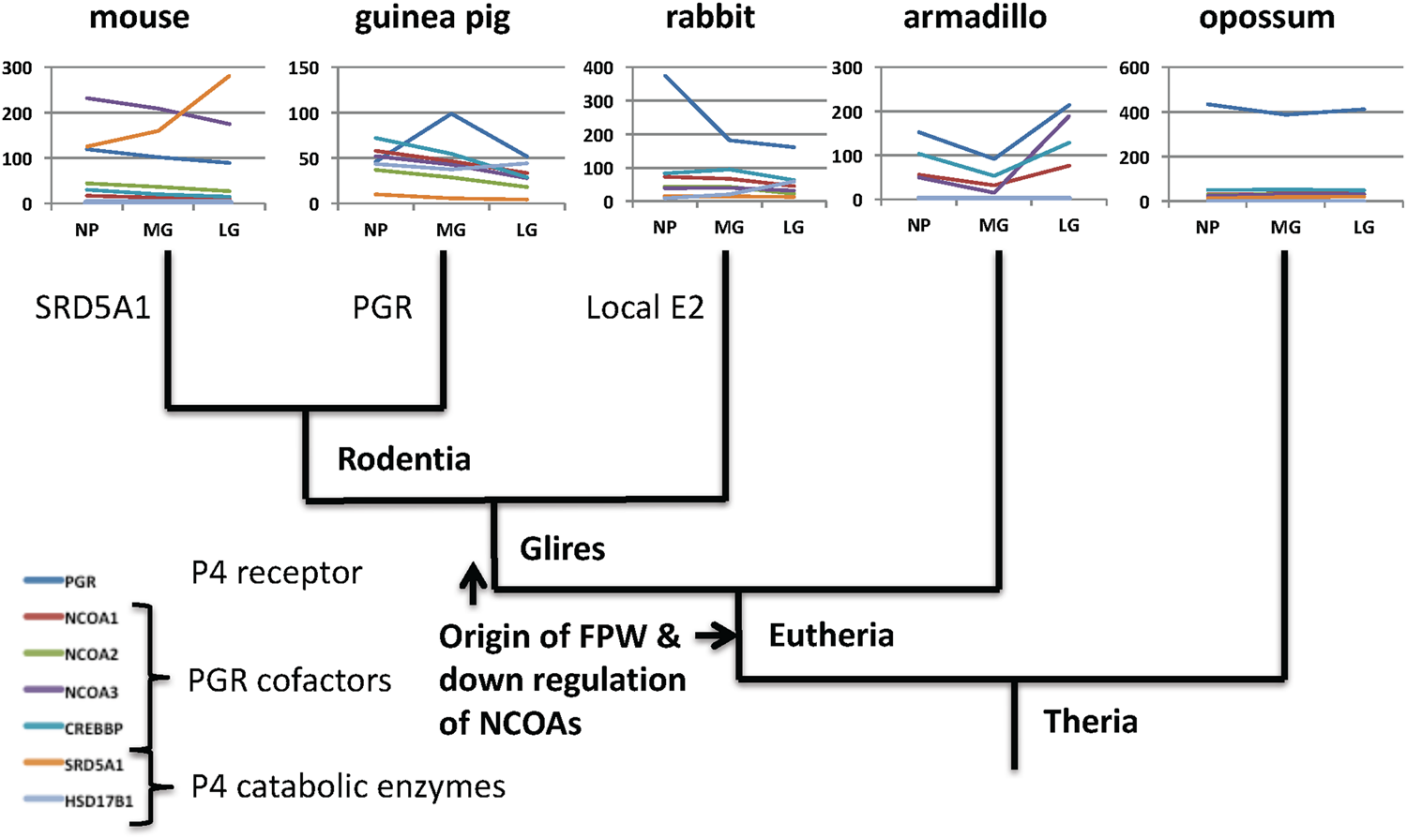
Evolutionary steps in the evolution of putative mechanisms of functional progesterone withdrawal. No modulation of genes related to steroid signaling is observed in opossum, while the expression of these genes is modulated in eutherian mammals suggesting that functional progesterone withdrawal evolved in the eutherian clade. The exact time of its evolution is not clear, because of incomplete data about the armadillo. It could be as early as the eutherian stem or the stem of Glires. Other putative mechanisms are species specific, with putative local estradiol synthesis in rabbit, *PGR* decrease in guinea pig and up-regulation of *SRD5A1* in mouse.

There is broad agreement that parturition and cervical ripening depend on a decrease in the level of P4 signaling^7, 8^. Some of the molecular mechanisms for functional progesterone withdrawal (FPW) have been worked out, in particular for the myometrium^44, 45^. In the mouse cervix it has been shown that the expression of the P4 catabolic enzyme 5α steroid reductase (HSD5A1) is essential for cervical ripening^21^. Our data also shows that *HSD5A1* mRNA expression is only found in the mouse (Fig. 2A), and not in the other eutherians examined (in armadillo this gene was not mapped). Other likely and species-specific mechanism for FPW are the down-regulation of PGR in guinea pig^39^. All eutherians, with the exception of guinea pig, have at least one paralog of the 20α HSD gene, called *AKR1C1* in humans, up-regulated towards term, which could indicate increased local breakdown of P4. But enzymes belonging to the AKR1C family are known to also have other enzymatic activities as well^27, 29^. In contrast, in the opossum all these genes are expressed, with the exception of HSD17B1, and remain at a constant expression level through gestation (Fig. 7). This suggests that none of the likely mechanisms of progesterone withdrawal examined here (P4 catabolism, PGR and PGR cofactor regulation) are active in the opossum, suggesting a lack of functional progesterone withdrawal in the opossum cervix. To understand the significance of this finding one has to review the role of P4 in marsupial pregnancy.

In both eutherians and in marsupials P4 causes uterine secretion, which plays a critical role in provisioning the early embryo^46^. In eutherians, P4 is also maintaining myometrial quiescence^18, 19, 24, 47–49^, and is essential for the maintenance of pregnancy throughout gestation. In many marsupials, including the opossum, the end of pregnancy is also characterized by an increase in systemic E2 and a decrease of P4, but experimental evidence does not support a role of these endocrine changes for birth timing and cervical ripening^49^. Opossum does not seem to have a need for functional progesterone withdrawal for initiating parturition, consistent with the gene expression data presented here (Figs 2, 3, 4 and 6) and previous experimental evidence. Our data, however, is consistent with experimental evidence for a role of relaxin in the preparation of the birth canal in opossum with a >8 fold increase of relaxin 1 receptor mRNA, *RXFP1*, during gestation, while in mouse RXFP1 expression is low and decreasing. There is also evidence for increased receptivity to prostaglandin signaling. This inference also suggests that the role of E2 and P4 in regulating cervical ripening evolved after the most recent common ancestor of marsupials and eutherian mammals, but the roles of relaxin and prostaglandins are older.

### Putative mechanisms of functional progesterone withdrawal are species specific

**Figure 8:**
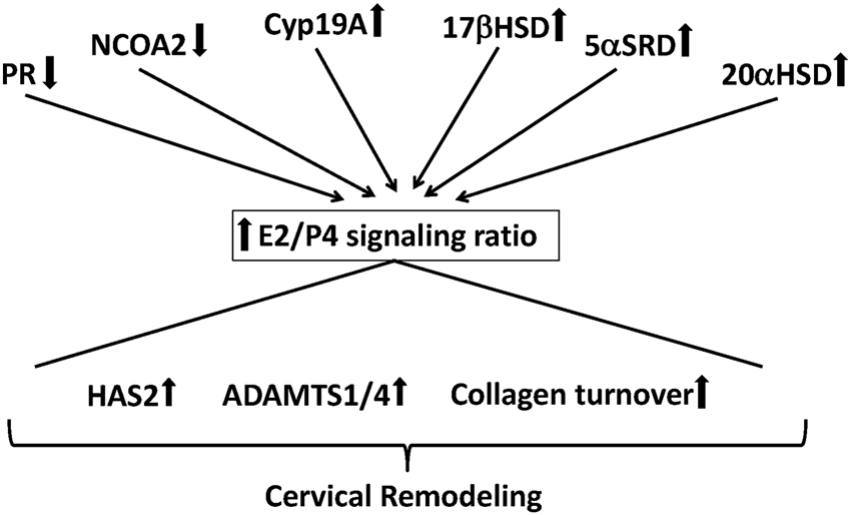
The “bow-tie architecture” of cervical remodeling explaining inter-species variation in gene expression. In this model it is assumed that there exists a conserved core regulatory mechanism controlling the expression of genes affecting the cervical extracellular matrix. This core mechanism is assumed to monitor the relative strength of E to P4 signaling and translates this signal into expression differences in ECM genes. Exactly which genes are regulated to change cervical ECM varies between species (not shown). In addition, genes that affect E and P4 signaling are different among species but all these different expression patterns likely increase the E/P4 signaling ratio towards term. The multiple ways to achieve the same outcome, E/P4 increase, can explain why the regulation of cervical ripening can differ from species to species.

Evidence for functional progesterone withdrawal is found in species with and without systemic progesterone withdrawal^20, 49^. For instance, in the mouse the end of pregnancy coincides with luteolysis and decreased systemic P4 levels. Never the less, both in the myometrium as well as the cervix experimental evidence shows functional progesterone withdrawal as well^20^. In our study we find that all eutherian species examined show changes in gene expression that are consistent with functional progesterone withdrawal (see above) and increased E2 production (rabbit). The rabbit is unique in that it does not show clear signs of progesterone withdrawal but expresses two key genes necessary for estradiol (E2) synthesis: CYP19A1, aka aromatase, and HSD17B1, which converts estrone into estradiol. In spite of all these differences, the available evidence is consistent with a model that cervical ripening towards term requires an increase in the ratio E/P4 signaling strength^16, 17, 49^ (Fig. 8). Hence our data is consistent with a model where a conserved core regulatory mechanism somehow is monitoring the E/P4 signaling strength ratio and translating this signaling ratio into changes in the extracellular matrix that ultimately leads to cervical compliance. It will be important to identify this core gene regulatory network. The species differences described in this paper likely represent different pathways towards achieving an increase in the E/P4 signaling ratio towards term (Fig. 8).

### The role of histone acetylation

The only broadly shared feature among eutherian species examined is a tendency to decrease the expression of nuclear receptor co-activators (NCOAs and CREBBP) towards the end of pregnancy. This finding is similar to earlier reports about the myometrium^43^. In the uterus proper, the decrease in NCOA expression is associated with a precipitous loss of acetylated histone-3 at the onset of labor. Systemic administration of histone deacetylase inhibitors (Trichostatin A) delayed parturition in mice^43^ and inhibits contractility in the human myometrium^50^. We also investigated H3 acetylation in the mouse cervix and found a similar drop in H3 acetylation with the onset of labor (Fig. 6A,B). These finding suggest a coordinated regulation of both, the myometrial contractility as well as cervical compliance and raises the possibility that influencing the activity of histone deacetylases could also affect the cervical ripening. The phylogenetic comparison further suggests that this mechanism is likely homologous at least among Glires or even eutherian mammals, i.e. evolved before the most recent common ancestor of eutherian mammals. If this is the case investigating this mechanism in a model organism (e.g. mouse) has a high chance of being translatable for human reproductive health as well.

## Conclusions

Our study identifies two features of cervical remodeling that are potentially conserved among eutherians: 1) an increase in the estrogen to progesterone signaling strength ratio and 2) massive histone deacetlylation at the onset of labor. The exact way how a shift in the E/P4 ratio is achieved, however, is highly species specific. We thus suggest that the most important open question is how estrogen and progesterone signaling interacts at the level of the cervical cells. Because of this conservation, model organism based research on this aspect of cervical ripening has the highest potential to be translatable into clinical outcomes in humans. Another interesting aspect is the role of histone deacetylation, although it still has to be established whether histone deacetylation plays a causal role in the control cervical remodeling rather than a consequence.

## Materials and Methods

### Animals

Opossum, *Monodelphis domestica*, specimen were obtained from animals bred in the inhouse colony according to approved IACUC protocols (2009–11313). Male and female animals are housed individually in different rooms. Breeding was initiated by exposing the female to male pheromones for two days and then housing with a male with video observation. After copulation was observed the animals were separated and tissue was harvested at 8 days post copulation and at day 14 post copulation. Armadillo samples were harvested from animals collected for the Yale Peabody Museum of Natural History in Centerville, TX, under protocol 2014–10906. Non-pregnant animals were collected in May, mid-gestational stages were obtained in the first week of January and late gestational stages at the beginning of March. Tissue harvesting from mouse, rabbit and guinea pig was done under Yale IACUC protocols 2012–11483 and 2015–11483. Timed pregnant rabbits were purchased from Charles River Laboratories and timed pregnant C57BL/6 mice from Harlan Spague Dawley Inc. The acquisition of guinea pig tissues is described in a previous paper^36^.

### Tissue harvesting

Cervix material was dissected from animals after euthanasia. The vaginal portion of the cervix was dissected and either preserved in RNA later for RNA sequencing or in 4% neutral para-formaldehyde for histological investigation and immuno-histochemistry. Non-pregnant samples were not staged for mouse and rabbit. Guinea pig samples were obtained during estrus as diagnosed by vaginal opening. Opossums were not cycling, i.e. not exposed to male pheromones and NP armadillo samples were harvested in early May, i.e. prior to the breeding season. Mid-gestation samples are from: mouse = 15 day post copulation (dpc), rabbit = 16dpc, guinea pig = 43dpc (for details see [36]), armadillo = harvested in the first week of January, which is about 4 to 6 weeks after implantation estimated to happen in mid to end November, opossum = 8dpc. Late gestation samples were obtained at: mouse = 18dpc, rabbit = 28dpc, guinea pig = 65dpc, armadillo = first week of March, opossumα= 14dpc. Tissue was collected in triplicate with few exceptions. For armadillo we were only able to harvest one individual in late gestation because of the limitations of field collecting and teaching schedules. For guinea pig the late gestation sample had four specimens. The latter was due to the need to have more animals since the exact day of parturition is not predictable. A total of 44 RNA seq. samples were analyzed for this study (Supplementary Table 1).

### Sequencing and data processing

RNA library preparation and high-throughput sequencing were performed on an Illumina HiSeq 2000 sequencing system following the protocol recommended by Illumina. Sequencing was done for each biological replicates at 1 × 75 bp strand specific by the Yale Center for Genome Analysis. Guinea pig sequence reads were aligned to the *Cavia porcellus* reference genome cavPor3.69, rabbit with oryCun2, armadillo data was re-analyzed with dasNov3, opossum monDom5, and mouse to GRCm38.69 assembly, using the splice junction mapper for RNA-seq reads TopHat2^51–53^. Sequencing depth for RNA-seq samples averaged 45 million reads per biological sample with >75% overall alignment rate. After alignment, read counts were determined with HTSeq-count v0.5.4p1 as described by the authors. Our guinea pig RNA-seq data was previously been deposited in GEO under accession number GSE47986. A complete list of accession numbers is provided in the supplementary material (Suppl. Table 2) and a plot of alignment statistics is provided in Suppl. Figs 3 and 4 and, and a table with the number reads and alignment statistics is given in Suppl. Table 3.

### Data Analysis

Relative RNA abundance was measured as Transcripts Per Million Transcripts (TPM) as recommended in refs 54 and 55. For all samples the alignment rate was at least 75%, and it was >80% for all armadillo samples with dasNov3 assembly. In order to mitigate the biases in comparative transcriptomic analyses, we took the following measures: 1) The mean of TPM values for all genes in a sample is proportional to the inverse of the number of genes used in the analysis. To account for the differences in the number of annotated genes between species, we TPM normalized read counts for all samples only using the genes with one-to-one orthologs between all species, thus making the TPM measurements as comparable between species as possible. 2) In addition, to circumvent the biases arising from differences in gene models between species (including aspects such as gene lengths), we avoid the comparisons of TPM measurements between species as far as possible. We measure differential expression of a given gene within species across stages of gestation, and compare the direction of differential expression between species e.g. upregulation in one species vs. downregulation in the other.

### Statistics

When correlations were calculated or differential expression was tested we transformed TPM data by square root transformation rather than the more widely used log-transformation for two reasons. First, TPM as well as RPKM data usually contains zero counts. In a log-transformation these data points will be transformed into minus infinity. In contrast $\sqrt{0}=0$. The second reason to prefer square root transformation is that it is in fact variance stabilizing^56^, while the log-transformation leads to an inflation of variance at low abundance values. Tests for differential expression were used to test specific hypotheses. For instance, we tested differences between stages for those genes that have been found differentially expressed in humans. For this reason, we did not use multiple comparison corrections. We used one way ANOVA and t-tests on square root transformed TPM data.

### Western Blotting

Cervix tissues of *M. musculus* were dissected. Nuclear proteins were extracted following the Pierce NE-PER Nuclear and Cytoplasmic Extraction Protocol (Thermo Scientific). Lysis buffers were supplemented with Halt Protease Inhibitor Cocktail (Thermo Scientific). All cell lysates were flash frozen and stored at −80C until use. After thawing, the protein concentrations were quantified using Pierce BCA protein assay kit (Thermo Scientific). Cell lysates were diluted in the lysis buffer to make a solution with 5 µg of protein. The solutions were combined with an equal volume of 2x loading buffer (2x NuPAGE LDS Sample buffer (Invitrogen) with 2x NuPAGE Sample Reducing Agent (Novex)), heated at 70 °C for 10 minutes, split in half to be loaded in two NuPAGE 4–12% Bis-Tris gels (Novex), and electrophoresed at 130 V for 60 minutes. Proteins were transferred to polyvinylidene difluoride membranes using the iBlot Gel Transer system (Invitrogen). Membranes were incubated with blocking buffer (3% bovine serum albumin in PBST) for 1 hour at room temperature, and then incubated with primary antibodies (Histone H3, 1:500 (Thermo Scientific #PA1–16941) or Acetyl Histone H3-Lys27, 1:1000 (Thermo Scientific #A16641)) overnight at 4 °C. After washing with PBST three times for 5 minutes, membranes were then incubated with the corresponding HRP-conjugated secondary antibody (goat anti-rabbit IgG HRP, 1:5000 (Santa Cruz #SC-2054)) for 1hr at 4 °C, followed by washing with PBST three times for 5 minutes. For signal detection, the membranes were incubated with Clarity Western ECL Substrate (Bio-Rad) for 5 minutes in the dark, and visualized with Bio-Rad Gel Doc System. After detection, membranes were stripped for 10 minutes in Restore Western Blot Stripping Buffer (Thermo Scientific) and reprobed with a loading control (Anti-Beta Actin Antibody [AC-15], 1:10,000 (Abcam #AB6276)), followed by secondary antibody (Donkey anti-mouse, 1:10,000 (Jackson Immuno Research Laboratories #715-035-150)).

### Immunohistochemistry

For the immuno-localization of NCOA2 we used frozen sections provided by the Condon Laboratory. For antigen retrieval, the slides were submerged in 0.3% sodium citrate solution and heated to 95 °C for 1 hour. The slides, still submerged, were subsequently cooled to 28 °C. Blocking was performed using a solution of 1% Bovine serum albumin in PBS. Each section was then treated with a peroxidase suppressor (DAKO, ref K4011) before the application of a primary antibody, specified below. The slides were incubated with primary antibody, diluted from 1/100 to 1/1000 in blocking solution, in a humidification chamber overnight at 4 °C. The next day, the slides were washed in PBS followed by blocking solution for 5 minutes each before the application of the secondary antibody, Labelled Polymer-HRP Anti-Rabbit (DAKO, ref K4011). After one more wash of PBS and blocking solution, each for five minutes, the secondary antibody was detected. The detection was done through an incubation of AEC (DAKO, ref K3463) and ultra-pure water for five minutes each. The first control used rabbit-IgG (Santa Cruz Biotech, sc-2027) as a primary antibody, while the second used no primary antibody. The procedure followed for the controls was otherwise identical to that of the experimental. NCOA2 was detected with rabbit antibody A300-346A from Bethyl raised against the human antigen and proven reactivity for human and mouse antigens.

### Ethics Statement

All experiments were performed according to all relevant guidelines and regulations. Specifically, for this study animals where only used for tissue harvesting and euthanized according to the guidelines provided by Yale IACUC. The relevant experimental protocols were approved by Yale IACUC under the protocol number #2015-11483 - “Evolution of gene expression of the uterine cervix and progesterone withdrawal among mammals”.

## Electronic supplementary material

Full Western Blotting Results

Suppementary Information
