# Supplementary material for: Evolution of Gene Expression in the Uterine Cervix related to Steroid Signaling: Conserved features in the regulation of cervical ripening
Source: Sci Rep. 2017 Jun 30;7:4439. doi: 10.1038/s41598-017-04759-6 (PMC5493687; doi:10.1038/s41598-017-04759-6)

Full western blotting results for "**Evolution of Gene Expression in the Uterine Cervix related to Steroid Signaling: Conserved features in the regulation of cervical ripening** "

Günter P. Wagner [1,2,3,5], Mauris C. Nnamani[1,3], Arun Rajendra Chavan[1,3], Jamie Maziarz[3], Stella Protopapas[3], Jennifer Condon[4,5] and Roberto Romero [4,5,6,7]

1) Department of Ecology and Evolutionary Biology, Yale University

2) Department of Obstetrics, Gynecology and Reproductive Science, Yale University

3) Yale Systems Biology Institute

4) Perinatology Research Branch, NICHD, NIH

5) Department of Obstetrics and Gynecology, Wayne State University

6) Department of Obstetrics and Gynecology, University of Michigan

7) Department of Epidemiology, Michigan State University

Inventory

- Experiment 1: 070516/1
- Experiment 2: 070516/2
- Experiment 3: 102115

Experiment 1: 070516/1

Histone 3 (gel #1)

Primary Antibody: Polyclonal Rabbit anti-Histone H3 (PA1-16941) 1:500

Secondary Antibody: goat anti-Rabbit IgG HRP (sc-2054) 1:5000

Predicted Size: 17kD

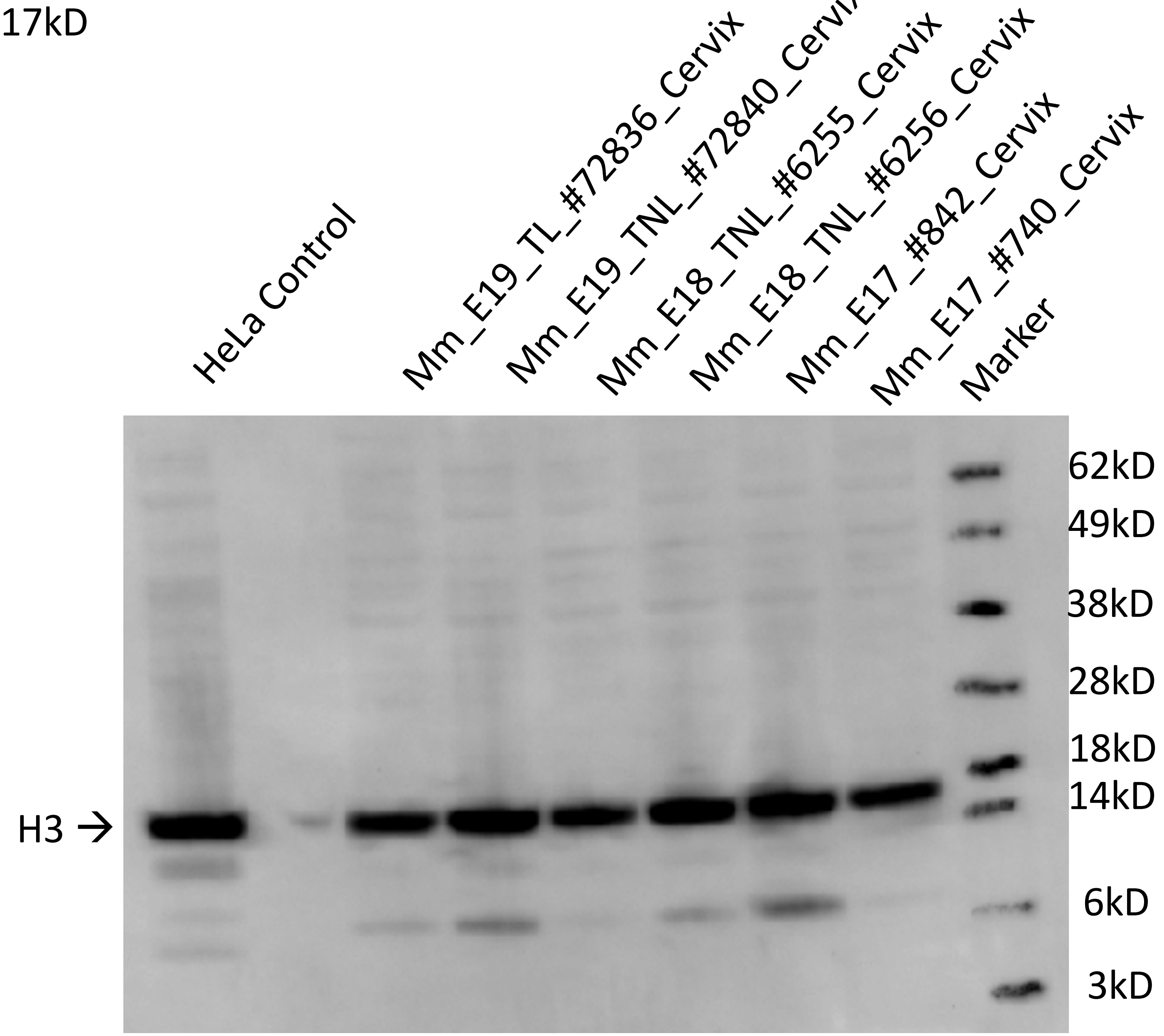

Histone 3 Stripped and Re-probed with Loading Control Actin (gel #1)
Primary Antibody: Monoclonal Mouse anti-beta Actin (AC-15)
Secondary Antibody: Peroxidase AffiniPure Donkey Anti-Mouse IgG (H+L) (Cat#715-035-150)
1:10,000
Predicted Size: 42kD

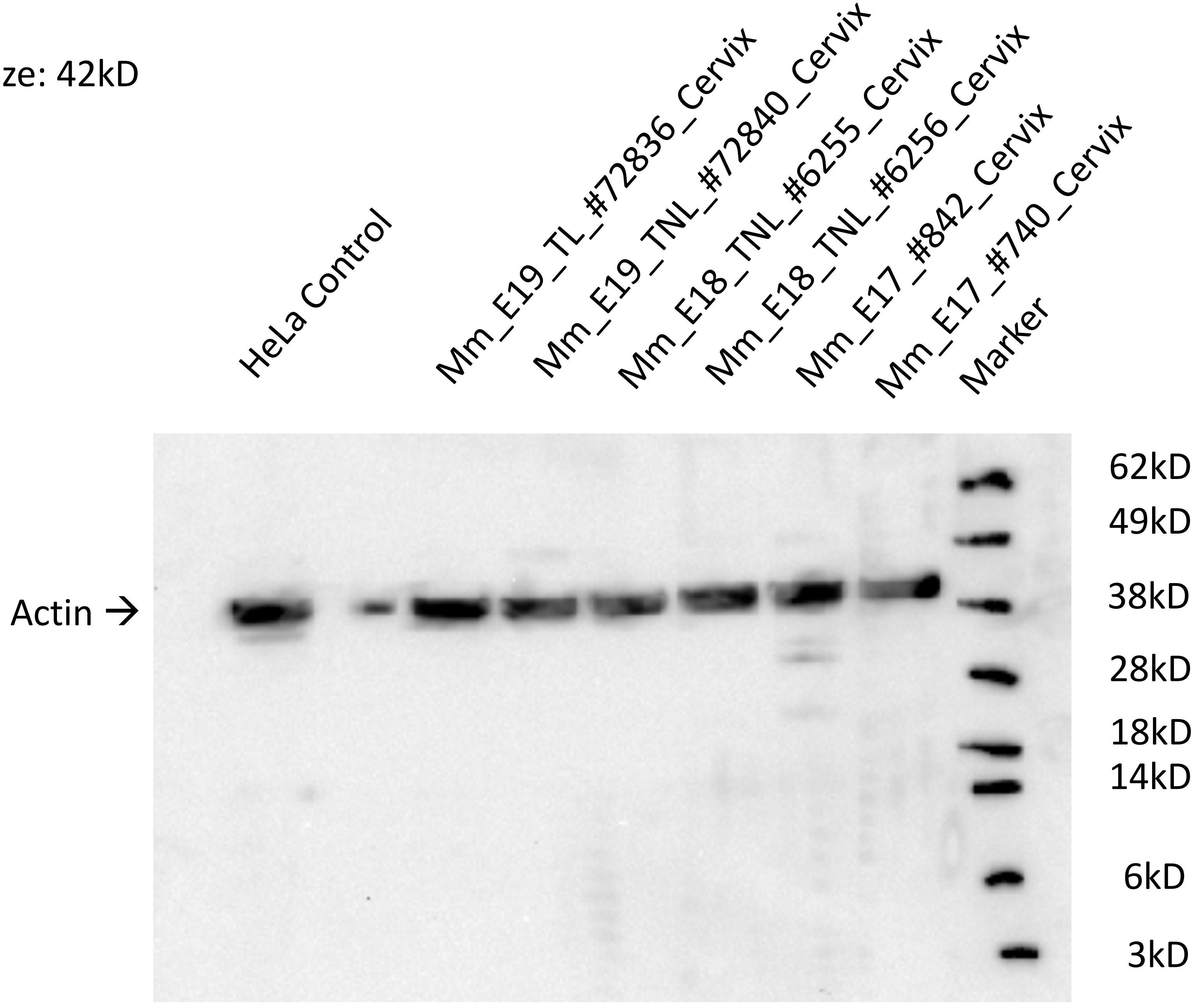

Acetyl Histone H3 Lys 27 (gel #1)

Primary Antibody: Polyclonal Rabbit anti-Acetyl Histone H3 Lys 27 (A16641) 1:1000

Secondary Antibody: goat anti-Rabbit IgG HRP (sc-2054) 1:5000

Predicted Size: 17kD

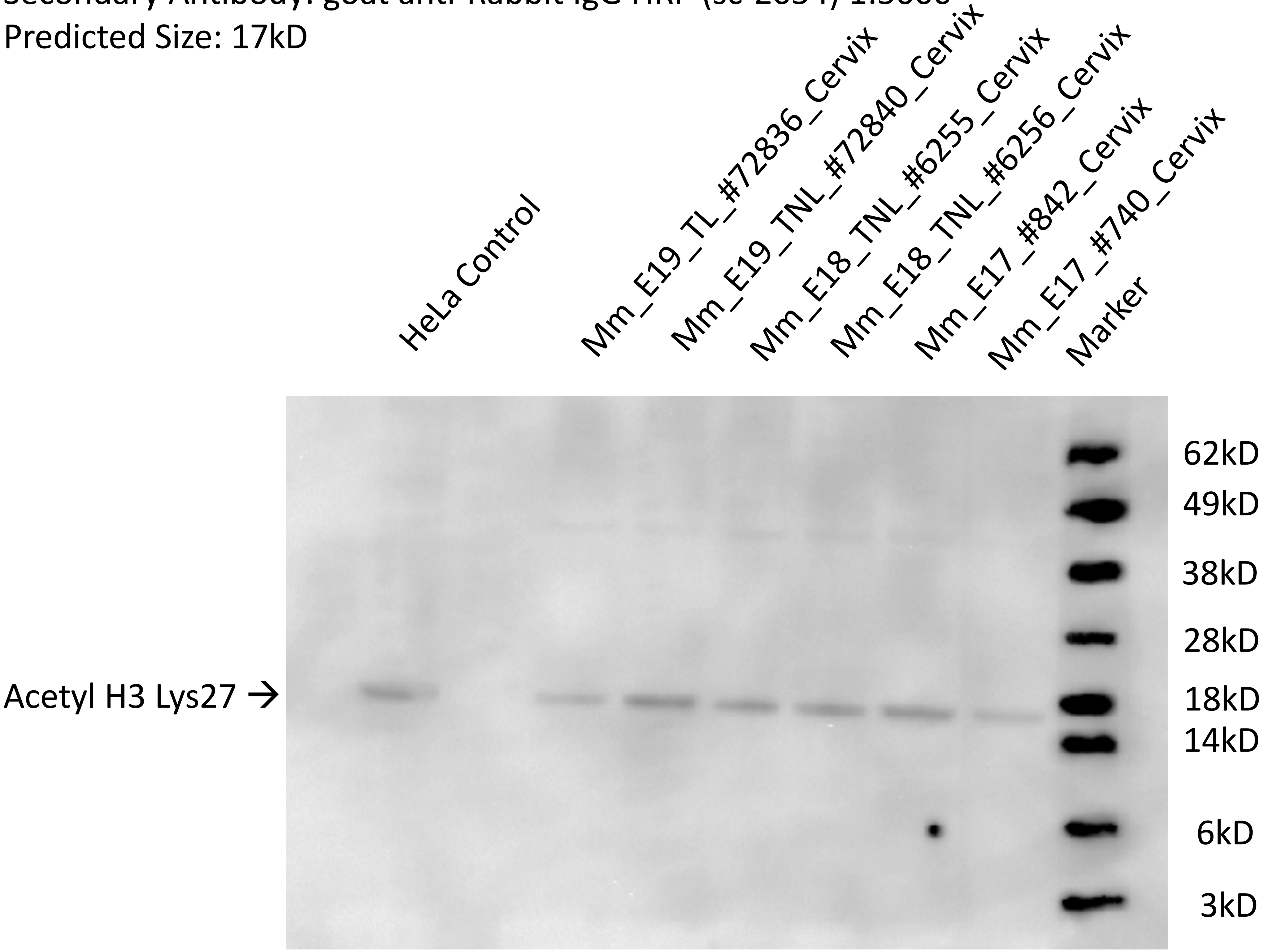

Acetyl Histone H3 Lys 27 Stripped and Re-probed with Loading Control Actin (Gel #1)
Primary Antibody: Monoclonal Mouse anti-beta Actin (AC-15)
Secondary Antibody: Peroxidase AffiniPure Donkey Anti-Mouse IgG (H+L) (Cat#715-035-150)
1:10,000
Predicted Size: 42kD

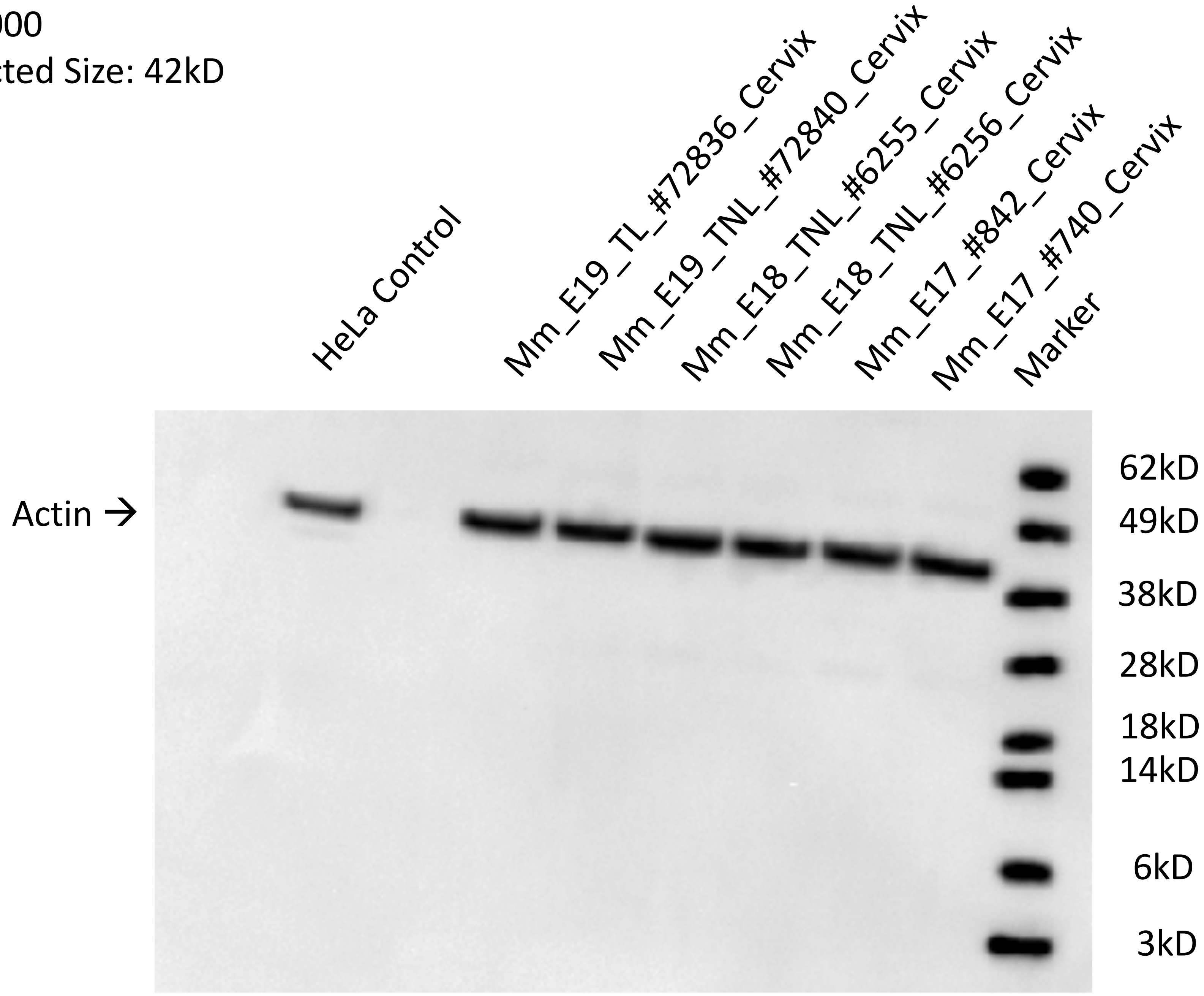

Experiment 2: 070516/2

Histone 3 (gel #2)

Primary Antibody: Polyclonal Rabbit anti-Histone H3 (PA1-16941) 1:500

Secondary Antibody: goat anti-Rabbit IgG HRP (sc-2054) 1:5000

Predicted Size: 17kD

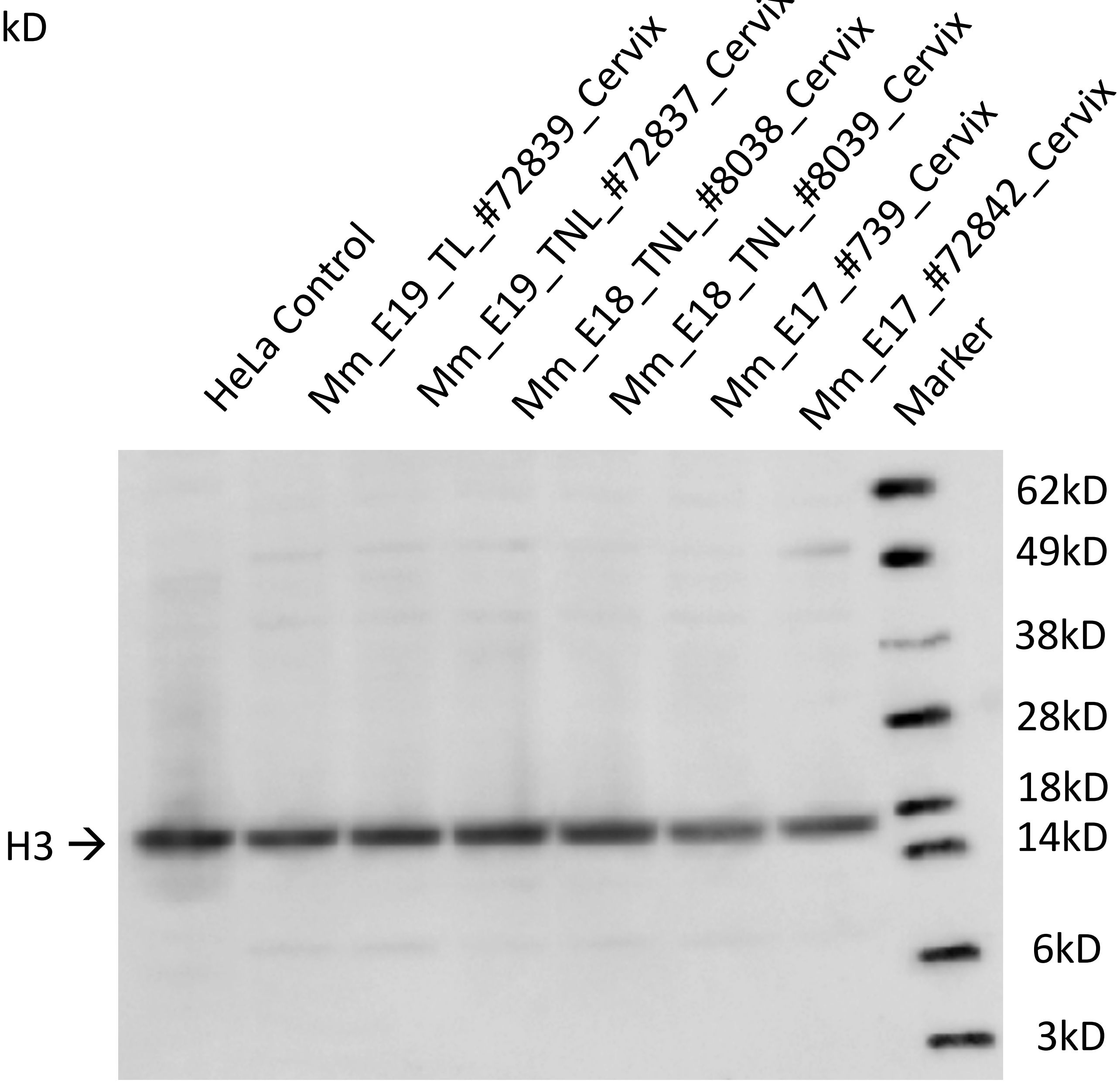

Histone 3 Stripped and Re-probed with Loading Control Actin (gel #2)

Primary Antibody: Monoclonal Mouse anti-beta Actin (AC-15)

Secondary Antibody: Peroxidase AffiniPure Donkey Anti-Mouse IgG (H+L) (Cat#715-035-150)

1:10,000

Predicted Size: 42kD

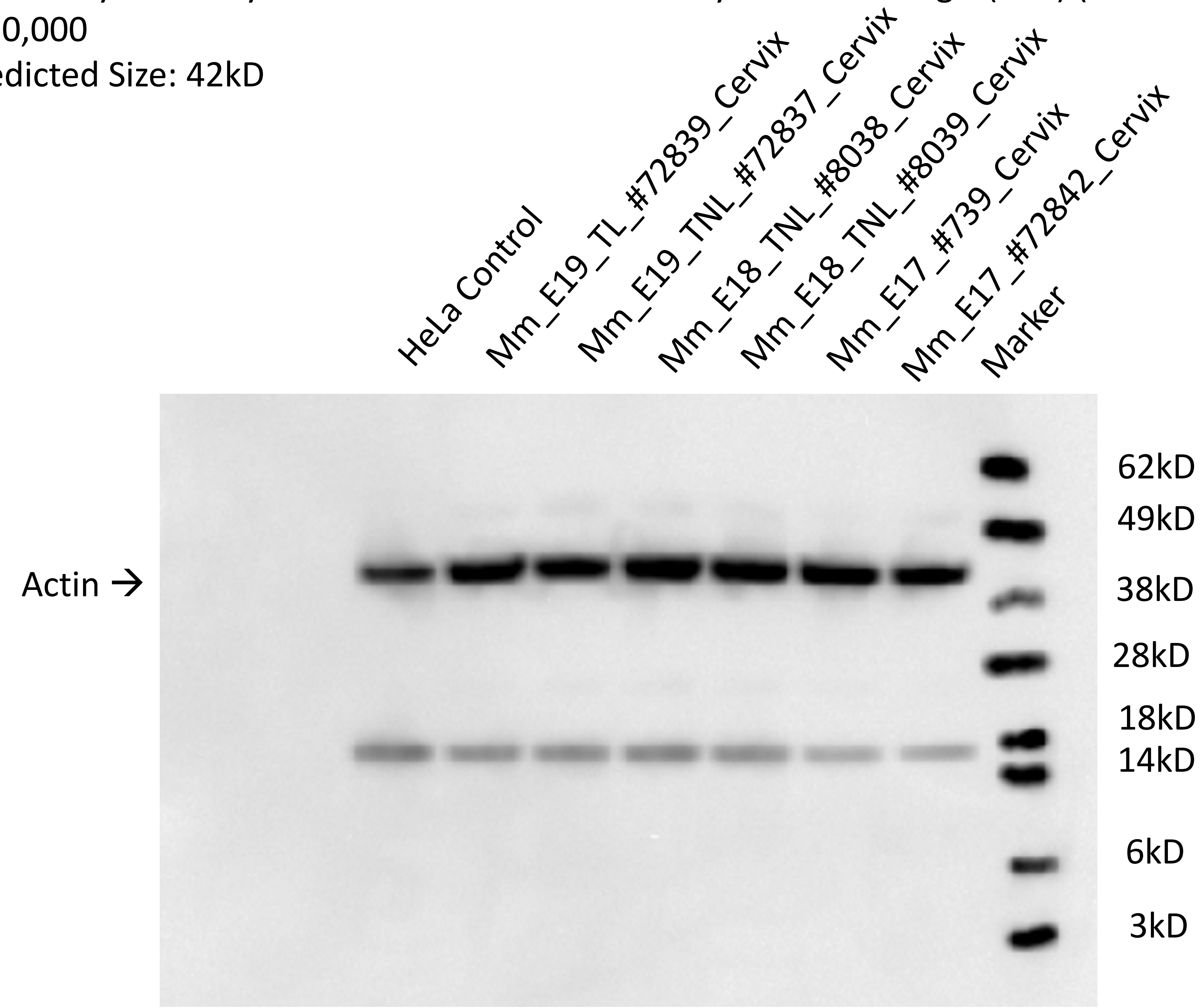

Acetyl Histone H3 Lys 27 (gel #2)

Primary Antibody: Polyclonal Rabbit anti-Acetyl Histone H3 Lys 27 (A16641) 1:1000

Secondary Antibody: goat anti-Rabbit IgG HRP (sc-2054) 1:5000

Predicted Size: 17kD

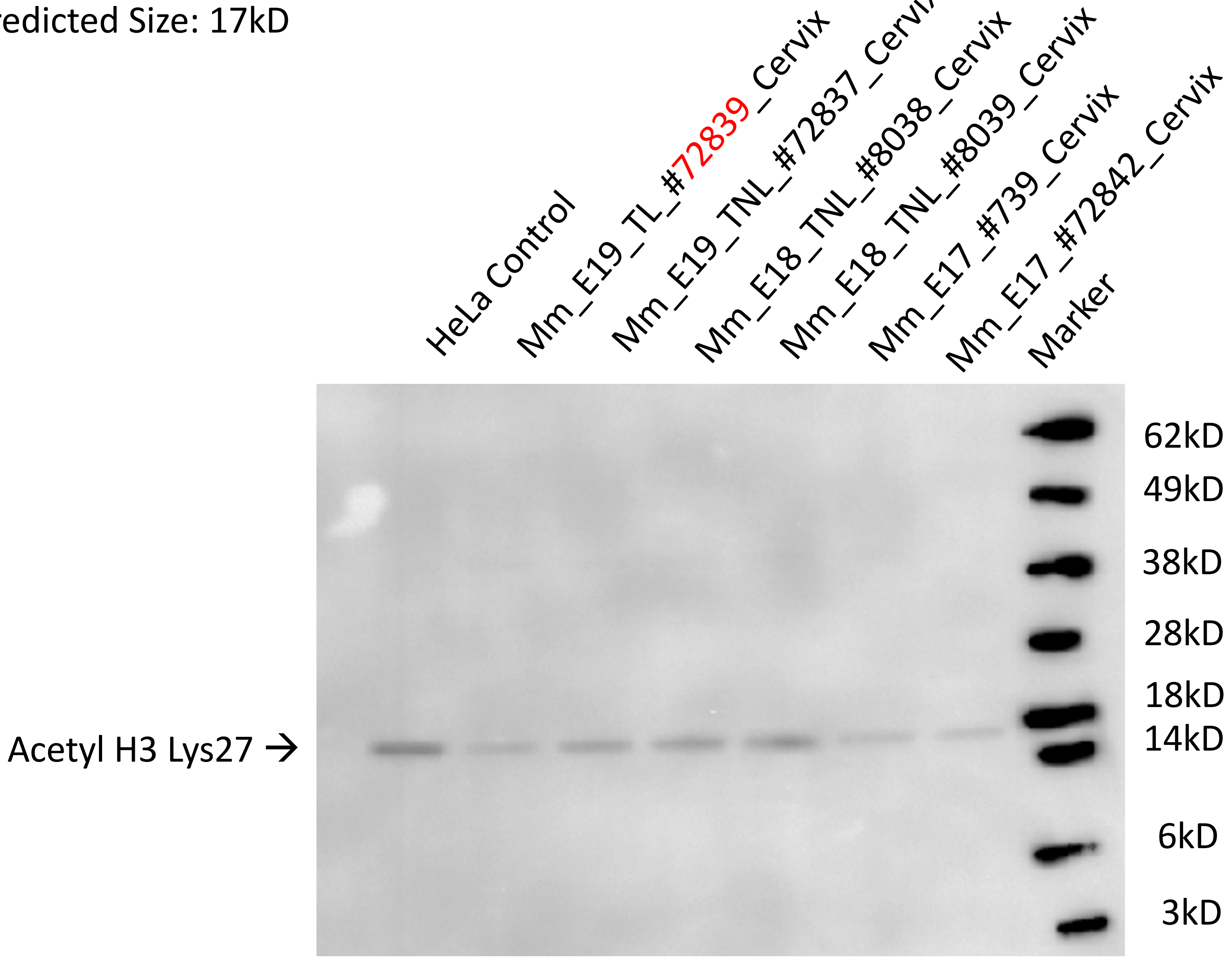

Acetyl Histone H3 Lys 27 Stripped and Re-probed with Loading Control Actin (Gel #2)
Primary Antibody: Monoclonal Mouse anti-beta Actin (AC-15)
Secondary Antibody: Peroxidase AffiniPure Donkey Anti-Mouse IgG (H+L) (Cat#715-035-150)
1:10,000
Predicted Size: 42kD

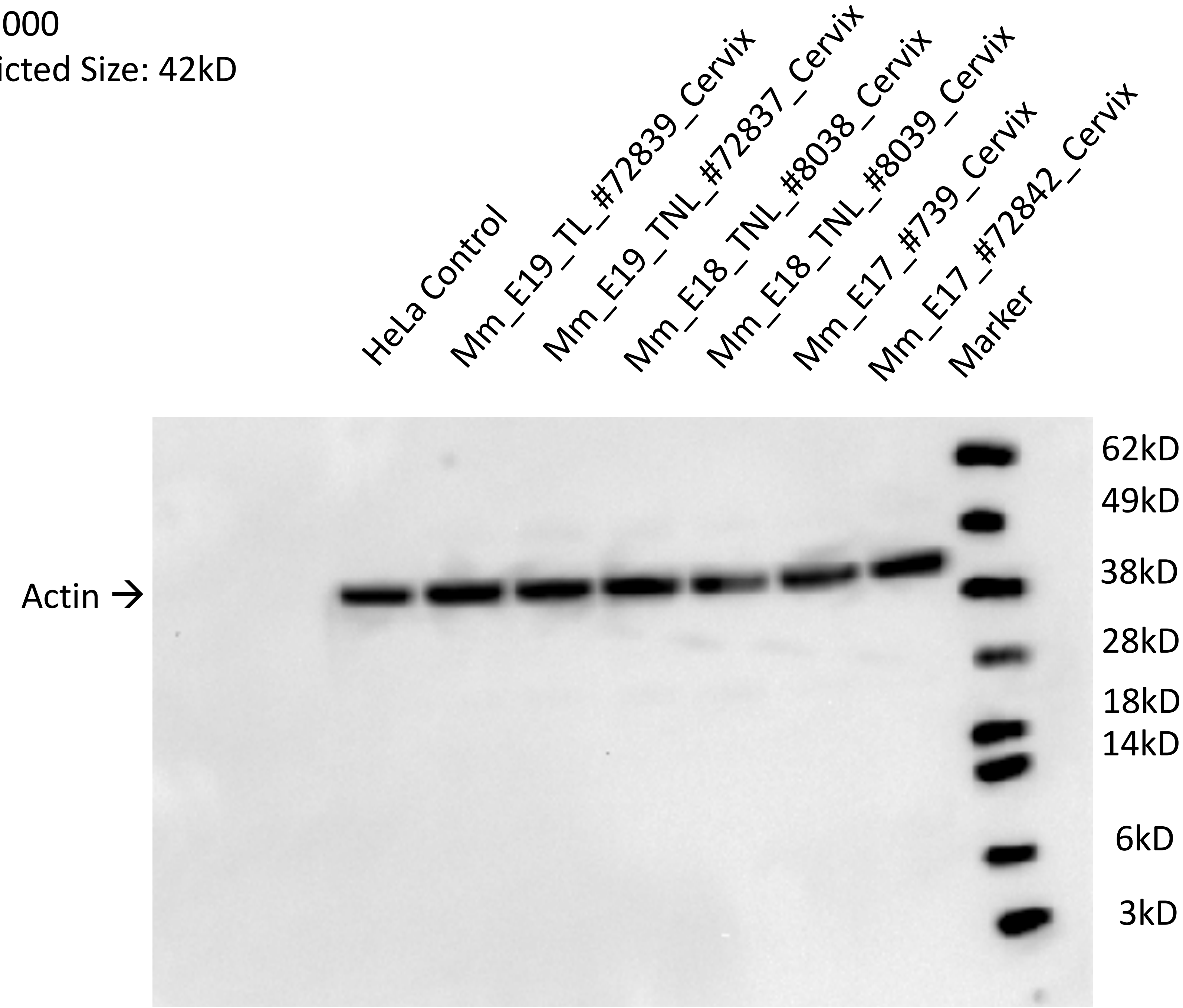

Experiment 3: 102115

Histone 3

Primary Antibody: Polyclonal Rabbit anti-Histone H3 (PA1-16941) 1:1000

Secondary Antibody: goat anti-Rabbit IgG HRP (sc-2054) 1:5000

Predicted Size: 17kD

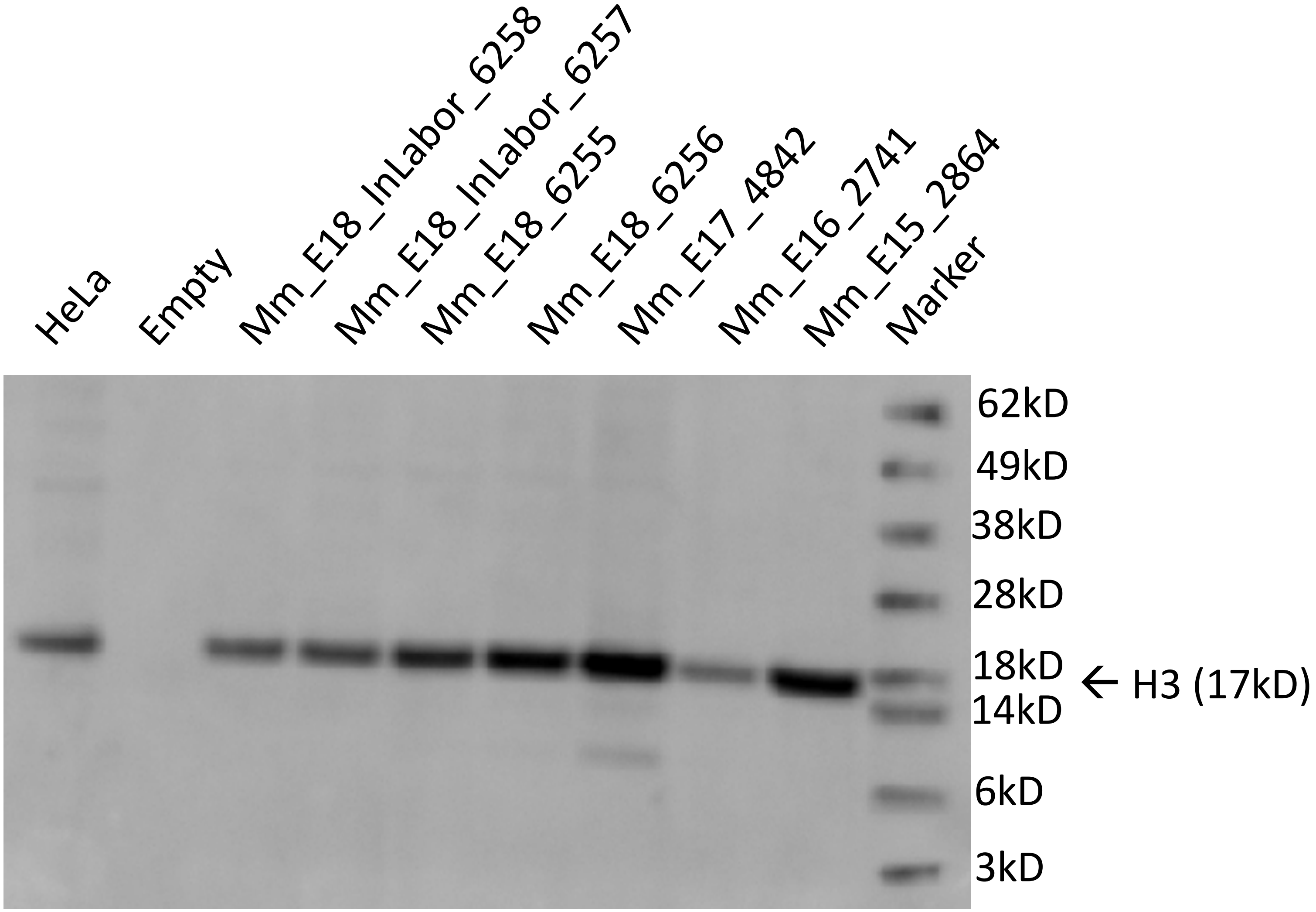

Histone 3 Stripped and Re-probed with Loading Control Actin

Primary Antibody: Monoclonal Mouse anti-beta Actin (AC-15)

Secondary Antibody: Peroxidase AffiniPure Donkey Anti-Mouse IgG (H+L) (Cat#715-035-150)

1:10,000

Predicted Size: 42kD

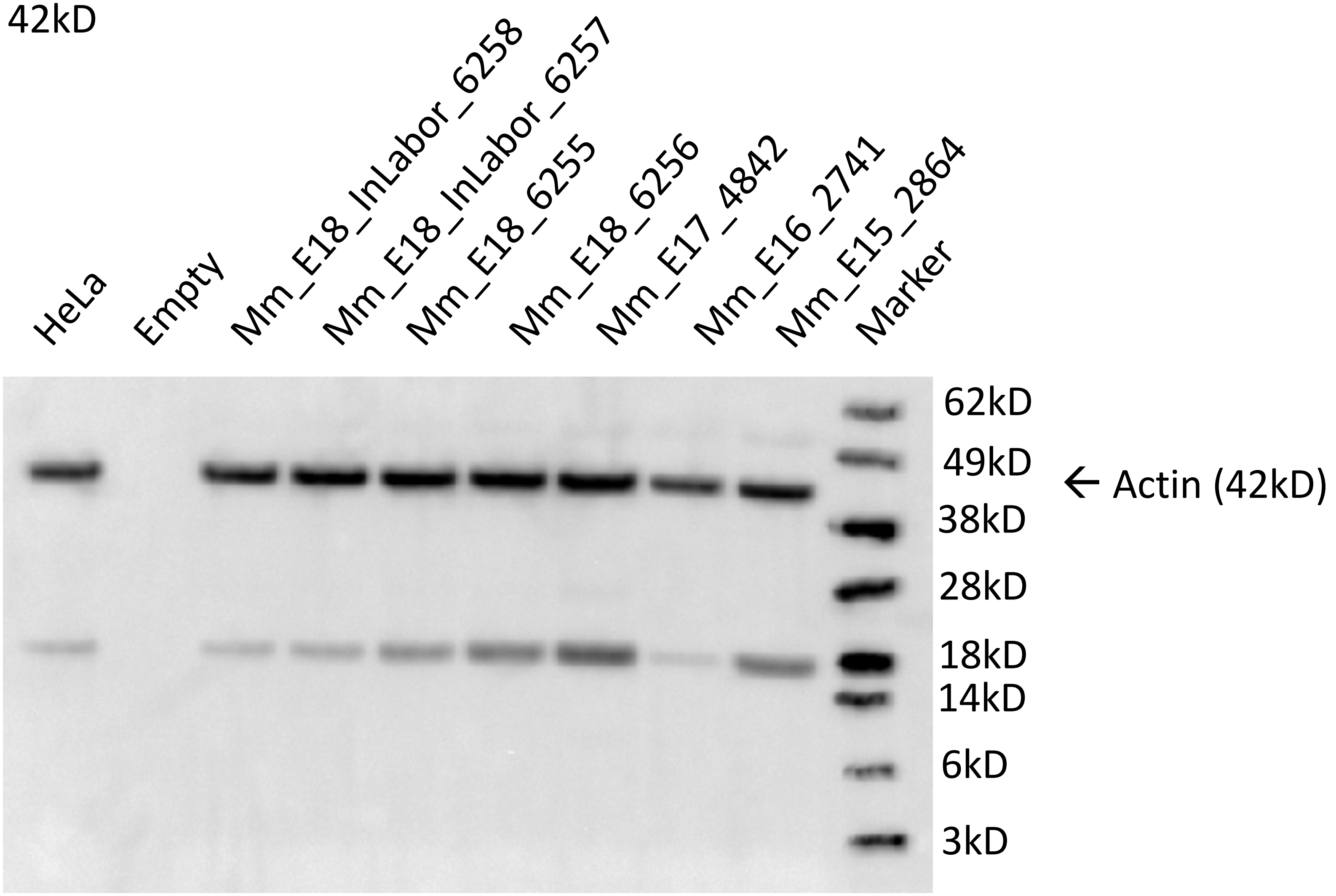

Acetyl Histone H3 Lys 27

Primary Antibody: Polyclonal Rabbit anti-Acetyl Histone H3 Lys 27 (A16641) 1:1000

Secondary Antibody: goat anti-Rabbit IgG HRP (sc-2054) 1:5000

Predicted Size: 17kD

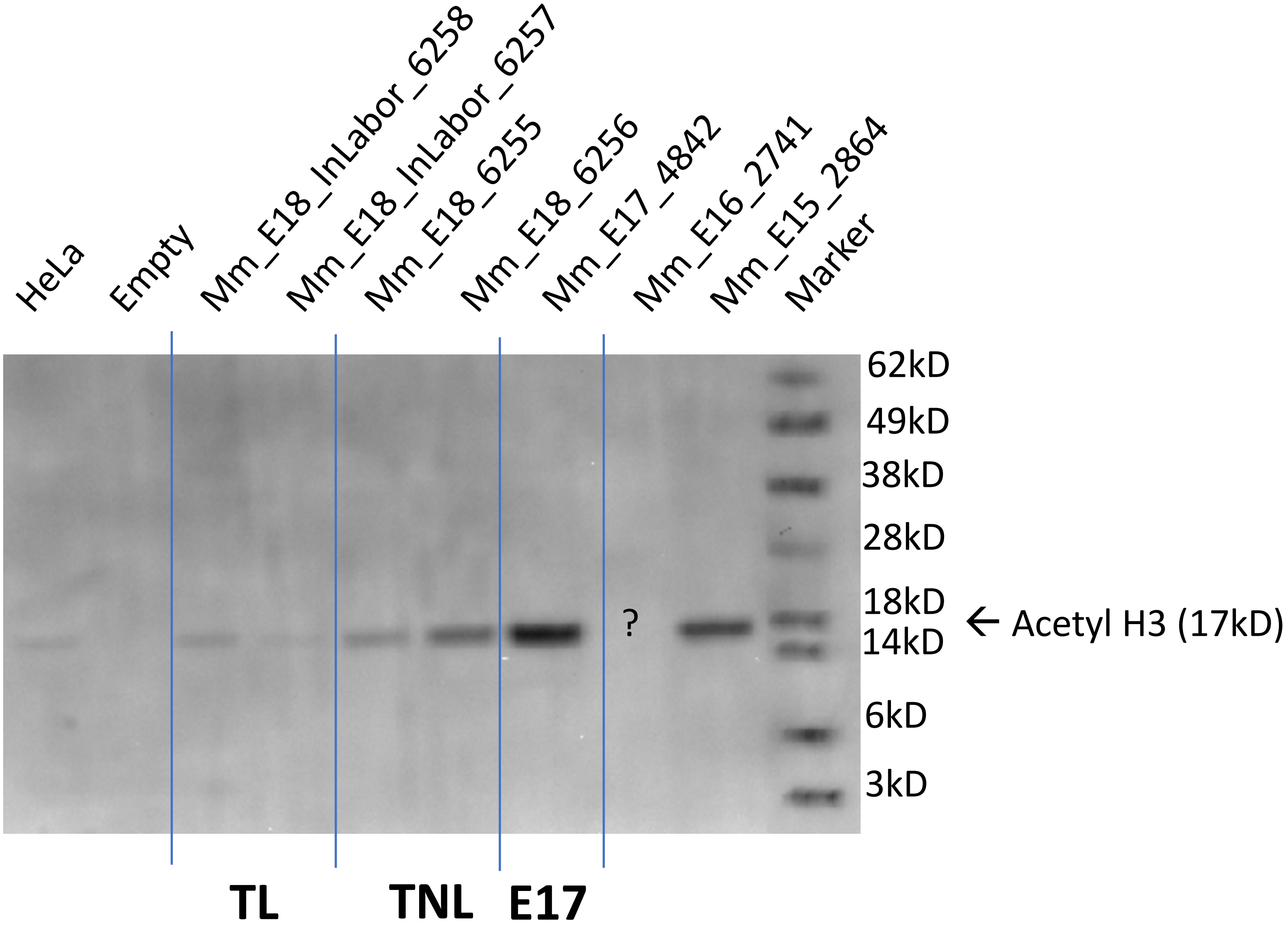

Acetyl Histone H3 Lys 27 Stripped and Re-probed with Loading Control Actin Gel #1

Primary Antibody: Monoclonal Mouse anti-beta Actin (AC-15)

Secondary Antibody: Peroxidase AffiniPure Donkey Anti-Mouse IgG (H+L) (Cat#715-035-150) 1:10,000

Predicted Size: 42kD

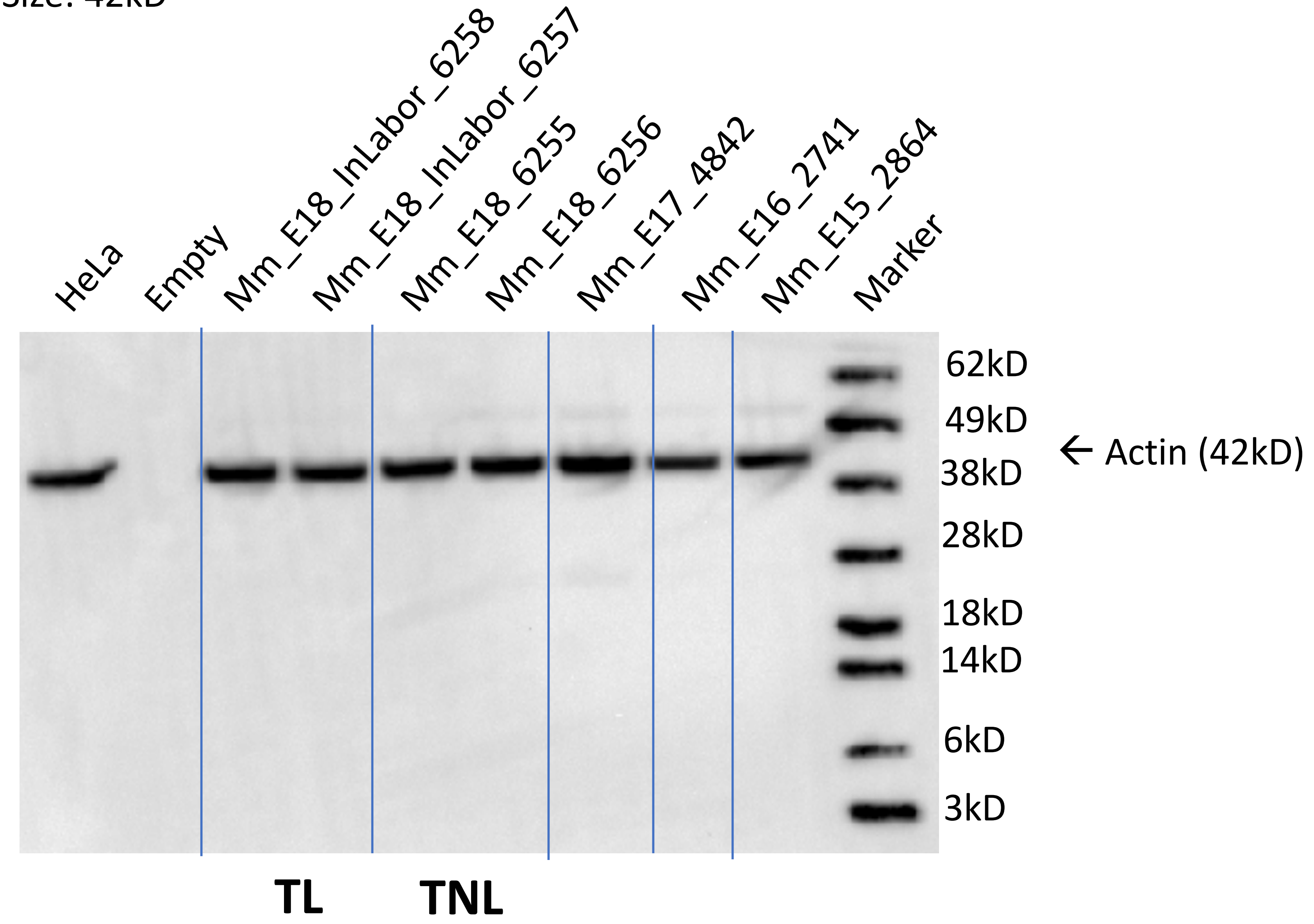

Supplement: Supplementary file 1 — Full Western Blotting Results [file 41598_2017_4759_MOESM1_ESM.pdf]
