# Supplementary material for: Evolution of Gene Expression in the Uterine Cervix related to Steroid Signaling: Conserved features in the regulation of cervical ripening
Source: Sci Rep. 2017 Jun 30;7:4439. doi: 10.1038/s41598-017-04759-6 (PMC5493687; doi:10.1038/s41598-017-04759-6)

Supplementary Material for Wagner et al., for

Evolution of Gene Expression in the Uterine Cervix related to Steroid Signaling: Conserved features in the regulation of cervical ripening

Günter P. Wagner [1,2,3,5], Mauris C. Nnamani[1,3], Arun Rajendra Chavan[1,3], Jamie Maziarz[3], Stella Protopapas[3], Jennifer Condon[4,5] and Roberto Romero [4,5,6,7]

1) Department of Ecology and Evolutionary Biology, Yale University
2) Department of Obstetrics, Gynecology and Reproductive Science, Yale University
3) Yale Systems Biology Institute
4) Perinatology Research Branch, NICHD, NIH
5) Department of Obstetrics and Gynecology, Wayne State University
6) Department of Obstetrics and Gynecology, University of Michigan
7) Department of Epidemiology, Michigan State University

Inventory:

Supplementary Table 1: number of samples analyzed

Supplementary Table 2: accession numbers for the data used in this study

Supplementary Table 3: read numbers and alignment statistics

Supplementary Figure 1: comparison of histone 3 acetylation between day 16 and day 18 TNL by Western Blotting.

Supplementary Figure 2: immunostaining for NCOA2 in the uterine stroma of the mouse cervix at day 10pc.

Supplementary Figure 3: percent reads aligned

Supplementary Figure 4: percent unique mapped reads

Supplementary Table 1: sample size by species and gestational stage entering the statistical analysis. In all cases were possible we collected at least three replicates. Lower numbers are either due to limitations of field collection, as for the armadillo, or because of sample quality controls, where some samples needed to be eliminated.

Species	NP	MG	LG	N
Opossum	3	2	3	8
Armadillo	4	5	1	10
Rabbit	3	3	3	9
Guinea pig	2	3	4	9
Mouse	2	3	3	8
total				44

Supplementary Table 2: Accession numbers of the RNA seq data utilized in this study.

Accession	Title	Release date	Status	Supplementary files
GSE85815	Evolution of Gene Expression in the Uterine Cervix related to Steroid Signaling: Conserved features in the regulation of cervical remodeling.	Aug 10, 2017	approved	
GSM2284926	Opossum Non-pregnant 2	Aug 10, 2017	approved	None
GSM2284927	Opossum Non-pregnant 3	Aug 10, 2017	approved	None
GSM2284928	Opossum Non-pregnant 4	Aug 10, 2017	approved	None
GSM2284929	Opossum Mid-gestation 1	Aug 10, 2017	approved	None
GSM2284930	Opossum Mid-gestation 2	Aug 10, 2017	approved	None
GSM2284931	Opossum Late-gestation 1	Aug 10, 2017	approved	None
GSM2284932	Opossum Late-gestation 2	Aug 10, 2017	approved	None
GSM2284933	Opossum Late-gestation 3	Aug 10, 2017	approved	None
GSM2284934	Armadillo Non-pregnant 1	Aug 10, 2017	approved	None
GSM2284935	Armadillo Non-pregnant 2	Aug 10, 2017	approved	None
GSM2284936	Armadillo Non-pregnant 3	Aug 10, 2017	approved	None
GSM2284937	Armadillo Non-pregnant 4	Aug 10, 2017	approved	None
GSM2284938	Armadillo Mid-gestation 1	Aug 10, 2017	approved	None
GSM2284939	Armadillo Mid-gestation 2	Aug 10, 2017	approved	None
GSM2284940	Armadillo Mid-gestation 3	Aug 10, 2017	approved	None
GSM2284941	Armadillo Mid-gestation 4	Aug 10, 2017	approved	None
GSM2284942	Armadillo Mid-gestation 5	Aug 10, 2017	approved	None
GSM2284943	Armadillo Late-gestation 1	Aug 10, 2017	approved	None
GSM2284944	Rabbit Non-pregnant 1	Aug 10, 2017	approved	None
GSM2284945	Rabbit Non-pregnant 2	Aug 10, 2017	approved	None
GSM2284946	Rabbit Non-pregnant 3	Aug 10, 2017	approved	None
GSM2284947	Rabbit Mid-gestation 1	Aug 10, 2017	approved	None
GSM2284948	Rabbit Mid-gestation 2	Aug 10, 2017	approved	None
GSM2284949	Rabbit Mid-gestation 3	Aug 10, 2017	approved	None
GSM2284950	Rabbit Late-gestation 1	Aug 10, 2017	approved	None
GSM2284951	Rabbit Late-gestation 2	Aug 10, 2017	approved	None
GSM2284952	Rabbit Late-gestation 3	Aug 10, 2017	approved	None
GSM2284953	Mouse Non-pregnant 2	Aug 10, 2017	approved	None
GSM2284954	Mouse Non-pregnant 3	Aug 10, 2017	approved	None
GSM2284955	Mouse Mid-gestation 1	Aug 10, 2017	approved	None
GSM2284956	Mouse Mid-gestation 2	Aug 10, 2017	approved	None
GSM2284957	Mouse Mid-gestation 3	Aug 10, 2017	approved	None
GSM2284958	Mouse Late-gestation 1	Aug 10, 2017	approved	None
GSM2284959	Mouse Late-gestation 2	Aug 10, 2017	approved	None
GSM2284960	Mouse Late-gestation 3	Aug 10, 2017	approved	None

Supplementary Table 3:

sample_name	species	stage	replicate_id	sample_type	total_reads	reads_kept	aligned	aligned_percent	single_feature	single_feature_percent_of_aligned
Cp_LG001	Guinea_Pig	LG	1	guinea_pig_LG	50612442	50468333	46709638	92.55236942	26326527	56.36208741
Cp_LG002	Guinea_Pig	LG	2	guinea_pig_LG	54258668	54165217	50056199	92.41391759	27881572	55.70053771
Cp_LG003	Guinea_Pig	LG	3	guinea_pig_LG	52945178	52874612	49403216	93.43466388	27752296	56.17507978
Cp_LG004	Guinea_Pig	LG	4	guinea_pig_LG	49811218	49733546	46533396	93.56540955	25804572	55.45387661
Cp_MG586	Guinea_Pig	MG	1	guinea_pig_MG	41335566	41226350	37878987	91.88052544	19109195	50.4480096
Cp_MG587	Guinea_Pig	MG	2	guinea_pig_MG	36821021	36742244	34146513	92.93529541	18730665	54.8538148
Cp_MG588	Guinea_Pig	MG	3	guinea_pig_MG	50213307	50077832	46287709	92.43153537	25380420	54.83187772
Cp_NP405	Guinea_Pig	NP	1	guinea_pig_NP	56316846	56208504	52066874	92.63166655	27351763	52.53198608
Cp_NP406	Guinea_Pig	NP	2	guinea_pig_NP	43093967	42981465	39518750	91.94370178	20047660	50.72948917
Cp_NP538	Guinea_Pig	NP	3	guinea_pig_NP	47016566	46896990	43194103	92.10421181	21988270	50.90572201
Dn_LG_1	Armadillo	LG	1	armadillo_LG	68249771	64392322	57088958	88.65802044	11241900	19.69189909
Dn_MG_1	Armadillo	MG	1	armadillo_MG	48608203	48352038	42771375	88.45826726	27248775	63.70797058
Dn_MG_2	Armadillo	MG	2	armadillo_MG	45788956	45555166	39985588	87.77399253	25272077	63.20296453
Dn_MG_3	Armadillo	MG	3	armadillo_MG	43019722	42810383	35797340	83.61835959	22147921	61.87029818
Dn_MG_4	Armadillo	MG	4	armadillo_MG	42341841	42136855	36557955	86.76004652	22708117	62.11539185
Dn_MG_5	Armadillo	MG	5	armadillo_MG	48165587	47909924	39060484	81.5290043	23667613	60.59221642
Dn_NP_1	Armadillo	NP	1	armadillo_NP	52835547	52774461	47324913	89.6738917	24574613	51.92743408
Dn_NP_2	Armadillo	NP	2	armadillo_NP	40878634	40845242	36380539	89.06922133	21371971	58.74561397
Dn_NP_3	Armadillo	NP	3	armadillo_NP	45693030	45641595	40388568	88.4907024	24426281	60.47820512
Dn_NP_4	Armadillo	NP	4	armadillo_NP	50912991	50845531	45522271	89.53052531	24286121	53.34997676
Md_LG_1	Opossum	LG	1	opossum_LG	39359083	39359077	35581084	90.40121546	22215799	62.43710563
Md_LG_2	Opossum	LG	2	opossum_LG	70224656	70224652	56173395	79.99099091	36141066	64.33840433
Md_LG_3	Opossum	LG	3	opossum_LG	36982414	36982405	32767953	88.60417001	21110343	64.42374658
Md_MG_1	Opossum	MG	1	opossum_MG	63128300	63128299	56301241	89.1854238	36972686	65.66939794

Md_MG_2	Opossum	MG	2	opossum_MG	61154932	60932911	55783717	91.54940423	34201505	61.31091085
Md_MG_3	Opossum	MG	3	opossum_MG	46809950	46809946	42915025	91.67928756	25844719	60.22300814
Md_NP_2	Opossum	NP	2	opossum_NP	32664767	32664766	29659237	90.79886566	18167445	61.25391897
Md_NP_3	Opossum	NP	3	opossum_NP	34851827	34851823	26150383	75.03304203	16159484	61.79444485
Md_NP_4	Opossum	NP	4	opossum_NP	44050076	44050075	38867700	88.23526407	23582958	60.67495118
Mm_LG_1	Mouse	LG	1	mouse_LG	54437863	54437861	52055295	95.62332914	45877140	88.13155319
Mm_LG_2	Mouse	LG	2	mouse_LG	49285708	49285706	46461118	94.26895092	40846286	87.91498732
Mm_LG_3	Mouse	LG	3	mouse_LG	58162495	58162486	53549919	92.06951539	46446271	86.73453082
Mm_MG_1	Mouse	MG	1	mouse_MG	49021199	49021198	46421534	94.69685747	40604614	87.46934989
Mm_MG_2	Mouse	MG	2	mouse_MG	75809913	75809910	71187697	93.9028908	62462058	87.74277106
Mm_MG_3	Mouse	MG	3	mouse_MG	40308192	40308191	37736900	93.62092186	32990444	87.42224189
Mm_NP_1	Mouse	NP	1	mouse_NP	89383559	89383554	83517929	93.43769101	72712248	87.06184273
Mm_NP_2	Mouse	NP	2	mouse_NP	32567324	32567323	31007922	95.2117618	27131130	87.49741437
Mm_NP_3	Mouse	NP	3	mouse_NP	52961678	52961677	49648919	93.74499036	43588407	87.79326495
Oc_LG_1	Rabbit	LG	1	rabbit_LG	46222920	46171097	39741651	86.07473849	20530367	51.65957247
Oc_LG_2	Rabbit	LG	2	rabbit_LG	67133148	67019698	57765575	86.19193569	29137515	50.44096765
Oc_LG_3	Rabbit	LG	3	rabbit_LG	42228095	42196747	36419642	86.30912236	17676374	48.53527665
Oc_MG_1	Rabbit	MG	1	rabbit_MG	38263569	38118535	32987373	86.53893178	17289620	52.41284294
Oc_MG_2	Rabbit	MG	2	rabbit_MG	50386895	50196035	43545916	86.75170459	23796229	54.64629335
Oc_MG_3	Rabbit	MG	3	rabbit_MG	33738960	33738957	29740721	88.14949733	13792305	46.37515345
Oc_NP_1	Rabbit	NP	1	rabbit_NP	43116639	42975763	37107245	86.34458683	18840043	50.77187218
Oc_NP_2	Rabbit	NP	2	rabbit_NP	49937933	49756522	42828961	86.0770795	20002199	46.70250815
Oc_NP_3	Rabbit	NP	3	rabbit_NP	47543944	47371272	41205713	86.98460324	18450808	44.77730552

stage: NP (non-pregnant), MG (mid-gestation), LG (late-gestation)

total_reads: total number of reads sequenced

reads_kept: reads used by tophat2 after removing low quality reads

aligned: number of reads that uniquely aligned to the genome

aligned_percent: percentage of reads_kept that uniquely aligned to the genome

single_feature: number of reads that were mapped uniquely to a gene feature by HTseq_count
single_feature_percent_of_aligned: percentage of aligned reads that were uniquely mapped to a gene feature by Htseq_count

inclusion: included or excluded from data analysis

Supplementary Figure 1: increase of histone 3 acetylation from day 16 to day 19 TNL as assessed by Western Blotting with an antibody for H3K27ac compared to actin loading control.

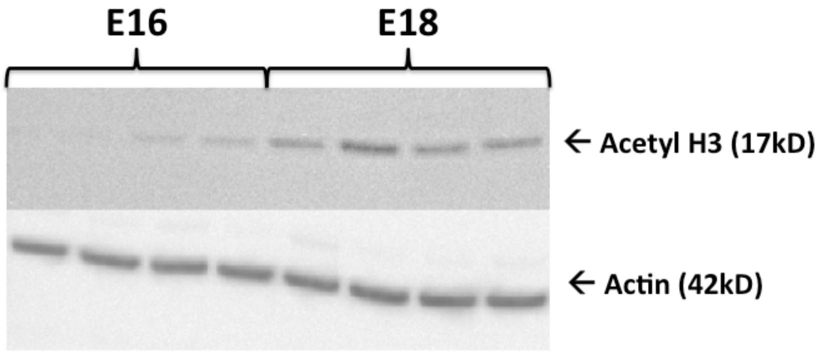

Suppl. Figure 1

Supplementary Figure 2: immno-histochemical localization of NCOA2 protein in the uterine part of the cervical stroma on day 19pc of the mouse. Note the contrast to the level of staining in the vaginal portion of the cervix as shown in Figure 5.

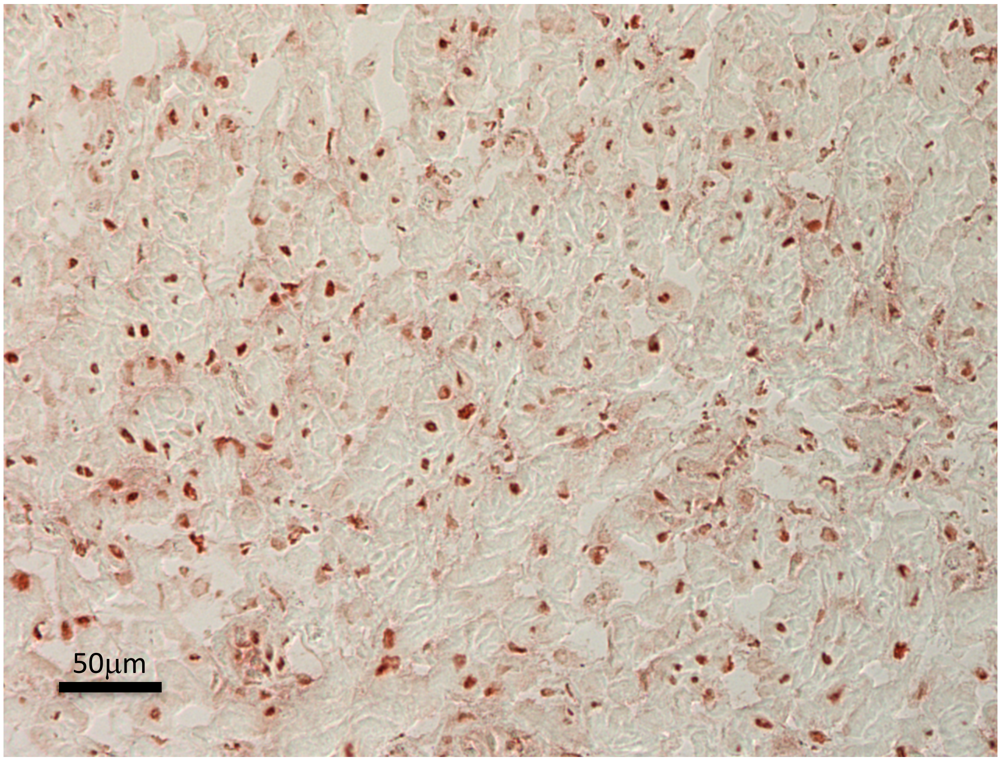

Suppl. Figure 2

Suppl. Figure 3: percent reads aligned

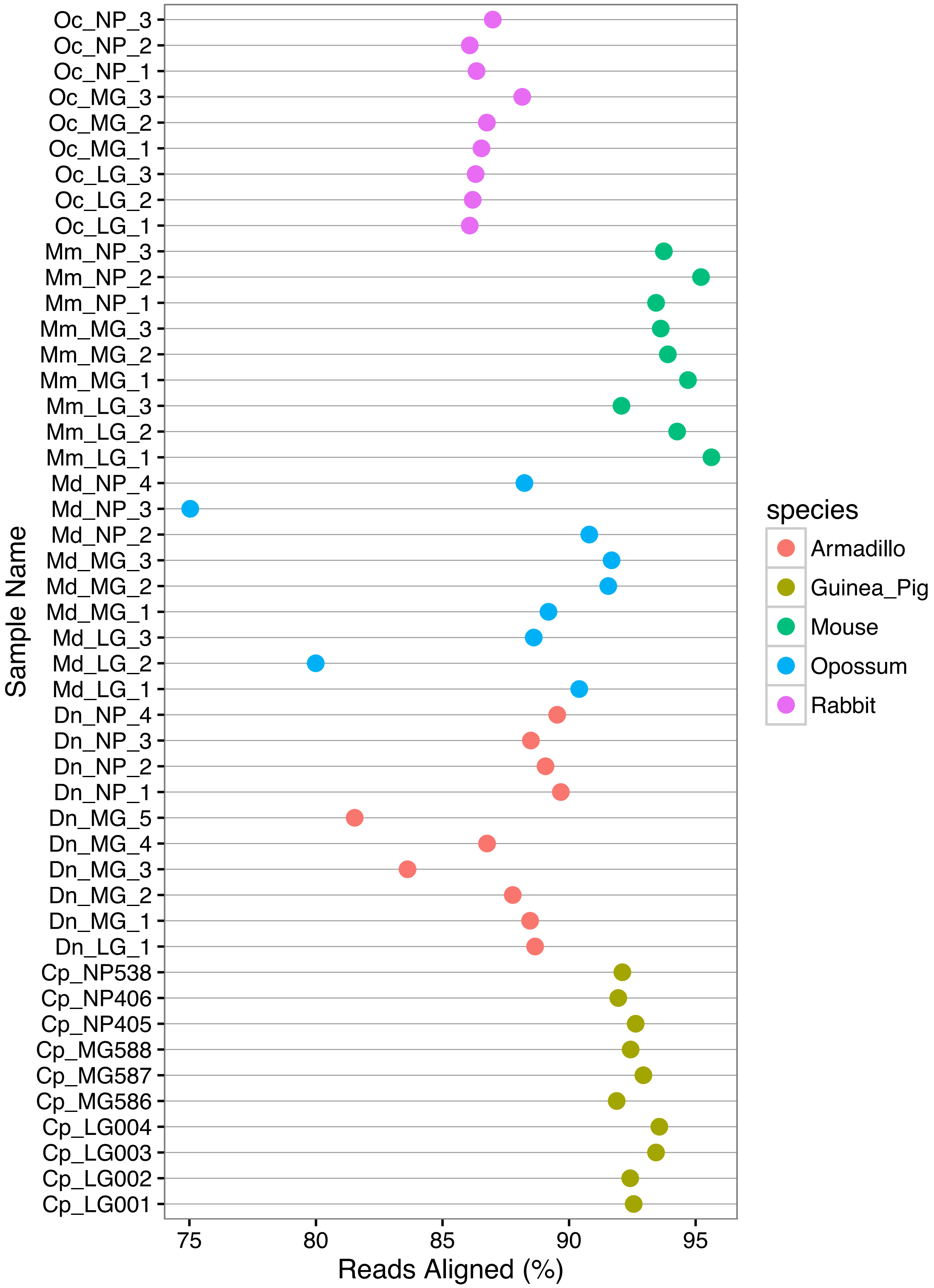

Suppl. Figure 2: percent unique mapped reads

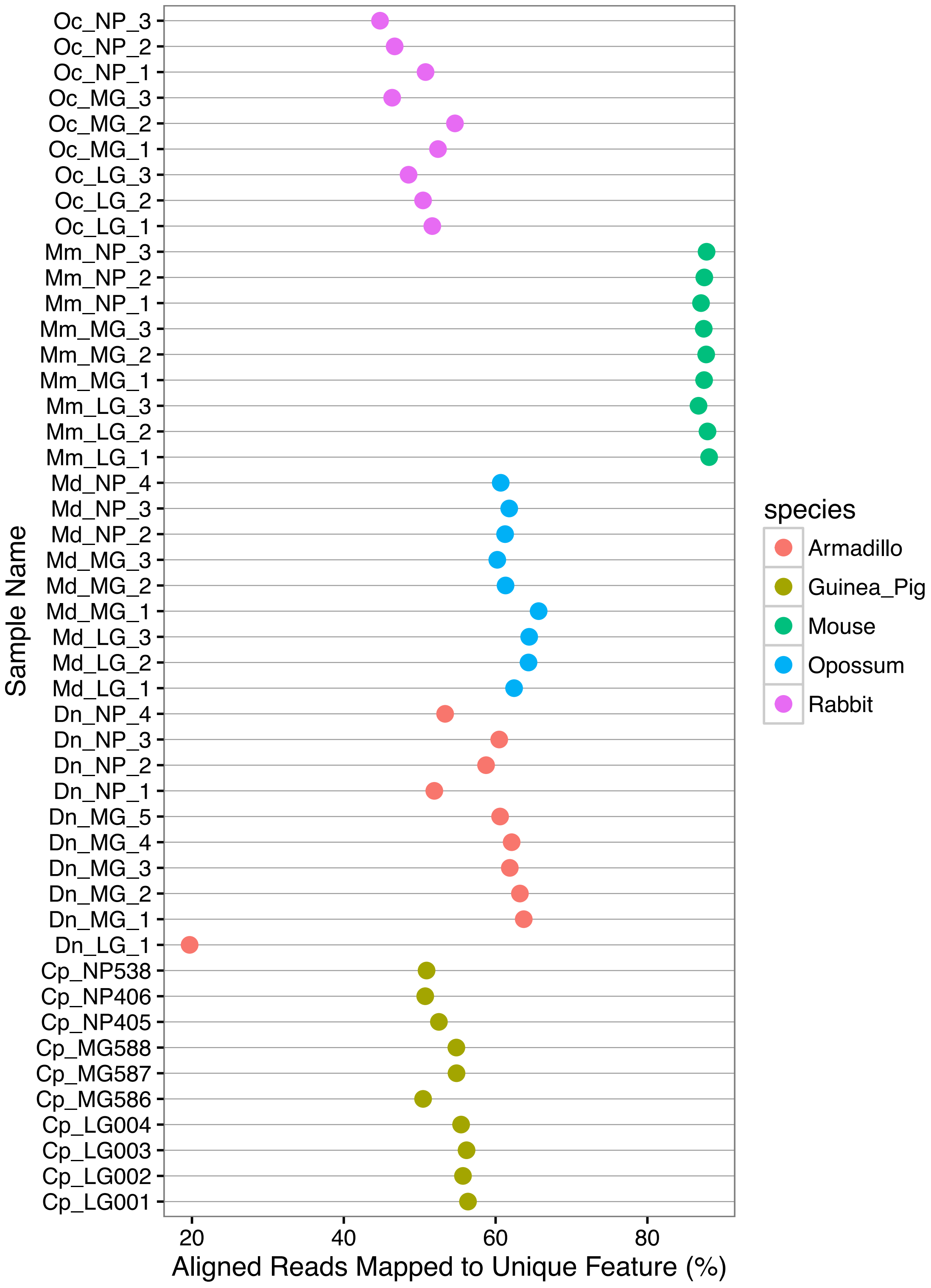

Supplement: Supplementary file 2 — Suppementary Information [file 41598_2017_4759_MOESM2_ESM.pdf]
